# EARLY STARVATION 1 Is a Functionally Conserved Protein Promoting Gravitropic Responses in Plants by Forming Starch Granules

**DOI:** 10.3389/fpls.2021.628948

**Published:** 2021-07-23

**Authors:** Kijong Song, Dae-Woo Lee, Jeongheon Kim, Jaewook Kim, Hwanuk Guim, Keunhwa Kim, Jong-Seong Jeon, Giltsu Choi

**Affiliations:** ^1^Department of Biological Sciences, Korea Advanced Institute of Science and Technology (KAIST), Daejeon, South Korea; ^2^Graduate School of Biotechnology and Crop Biotech Institute, Kyung Hee University, Yongin-si, South Korea; ^3^Research Center for Materials Analysis, Korea Basic Science Institute, Daejeon, South Korea

**Keywords:** Arabidopsis, gravitropism, liverwort, rice, starch, *EARLY STARVATION 1*

## Abstract

Starch granules in the endodermis of plant hypocotyls act as statoliths that promote hypocotyl negative gravitropism—the directional growth of hypocotyls against gravity—in the dark. To identify the molecular components that regulate hypocotyl negative gravitropism, we performed a mutagenesis screen and isolated *reduced gravitropic 1* (*rgv1*) mutants that lack starch granules in their hypocotyl endodermis and show reduced hypocotyl negative gravitropism in the dark. Using whole genome sequencing, we identified three different *rgv1* mutants that are allelic to the previously reported *early starvation 1* mutant, which is rapidly depleted of starch just before the dawn. *ESV1* orthologs are present in starch-producing green organisms, suggesting *ESV1* is a functionally conserved protein necessary for the formation of starch granules. Consistent with this, we found that liverwort and rice *ESV1* can complement the Arabidopsis *ESV1* mutant phenotype for both starch granules and hypocotyl negative gravitropism. To further investigate the function of *ESV1* in other plants, we isolated rice *ESV1* mutants and found that they show reduced levels of starch in their leaves and loosely packed starch granules in their grains. Both Arabidopsis and rice *ESV1* mutants also lack starch granules in root columella and show reduced root gravitropism. Together, these results indicate *ESV1* is a functionally conserved protein that promotes gravitropic responses in plants via its role in starch granule formation.

## Introduction

As sessile organisms, plants need to adjust their growth and development in response to various environmental factors. Gravity is one of these environmental factors that determines the growth orientation of plants via a process called gravitropism. Roots show positive gravitropism when they grow in the direction of gravity, while shoots including hypocotyls show negative gravitropism by growing in the direction opposite to gravity (Knight, 1806; Kiss, 2000). Plant gravitropic responses can be conceptually divided into four steps: gravity sensing, cellular signal formation, intercellular signal transmission, and asymmetric organ growth (Morita and Tasaka, 2004; Morita, 2010; Hashiguchi et al., 2013; Nakamura et al., 2019). Plants sense gravity via the directional sedimentation of amyloplasts, which are plastids filled with starch granules (Sack, 1991; Toyota et al., 2013). These amyloplasts move with gravity inside statocytes, gravity-sensing cells that include the columella cells in roots and the endodermal cells in hypocotyls and stems (Hashiguchi et al., 2013; Nakamura et al., 2019). The directional sedimentation of these amyloplasts induces an asymmetric localization of auxin efflux carriers including PIN-FORMED 3 (PIN3) in the direction of gravity in the presence of LAZY family proteins (Friml et al., 2002; Yoshihara and Iino, 2007; Harrison and Masson, 2008; Kleine-Vehn et al., 2010; Rakusova et al., 2011; Yoshihara et al., 2013; Taniguchi et al., 2017; Yoshihara and Spalding, 2017). Asymmetric localization of PIN3, in turn, produces an increasing auxin concentration gradient in the direction of gravity. In response to this asymmetric distribution of auxin, plant organs elongate asymmetrically and bend either toward or against gravity. Amyloplast sedimentation does not occur via a free-falling process. In stems, endodermal cells contain a large central vacuole that limits the movement of amyloplasts to trans-vacuolar strands and to the narrow spaces between the vacuolar and plasma membranes (Saito et al., 2005). Such constriction makes proper vacuolar membrane dynamics essential for shoot gravitropism. Indeed, several *shoot gravitropism* mutants that are defective in vacuolar membrane dynamics show abnormal localization and sedimentation of amyloplasts (Kato et al., 2002; Morita et al., 2002; Yano et al., 2003; Hashiguchi et al., 2014).

Starch synthesis occurs in plastids with the supply of ADP-Glucose (ADP-Glc) (Zeeman et al., 2010; Streb and Zeeman, 2012). In photosynthetic cells like Arabidopsis leaf mesophyll cells, fructose 6-phosphate (Fru-6-P) produced in the Calvin-Benson cycle is converted to glucose 6-phosphate (Glc-6-P) by plastidic phosphoglucoisomerase (PGI) (Yu et al., 2000). This, in turn, is converted to glucose 1-phosphate (Glc-1-P) by phosphoglucomutase (PGM) in plastids (Caspar et al., 1985). In non-photosynthetic cells like Arabidopsis hypocotyl endodermal cells and root columella cells, sucrose is metabolized to produce Glc-6-P in the cytosol (Slewinski and Braun, 2010). Cytosolic Glc-6-P is then transported into plastids by Glc-6-P/Phosphate translocator (GPT) and converted to Glc-1-P by PGM (Kofler et al., 2000; Fischer and Weber, 2002; Niewiadomski et al., 2005; Kunz et al., 2010). In the plastids of both photosynthetic and non-photosynthetic cells, Glc-1-P is converted to ADP-Glc by ADP-glucose pyrophosphorylase (AGPase). In cereal endosperm, however, Glc-1-P is converted to ADP-Glc by cytosolic AGPase and transported into the plastids (Jeon et al., 2010; Tuncel and Okita, 2013). ADP-Glc in plastids is then used for starch synthesis. Glucan chains are branched by branching enzymes (BE) and branching patterns are further reorganized by debranching enzyme (DBE). Linear (amylose) and highly branched (amylopectin) glucan chains are then packed in multiple layers of semi-crystalline and amorphous structures to form large starch granules (Zeeman et al., 2010). Starch granules in plastids are degraded by several enzymes (Smith et al., 2005; Streb and Zeeman, 2012). Starch degradation is initiated by the transient phosphorylation of glucosyl residues on the surface of amylopectin at the C_6_ and C_3_ positions by glucan, water dikinase (GWD) and phosphoglucan, water dikinase (PWD), respectively (Lorberth et al., 1998; Ritte et al., 2002, 2006; Baunsgaard et al., 2005; Kotting et al., 2005). This phosphorylation loosens the semi-crystalline structure of amylopectins on the surface of starch granules, making it accessible to other enzymes (Hejazi et al., 2008, 2009).

Phytochromes (phyA to phyE) are red and far-red light receptors promoting light responses (Legris et al., 2019). The phytochromes accomplish this in part via their inhibition of Phytochrome Interacting Factors (PIFs), a group of bHLH transcription factors (Leivar and Quail, 2011; Lee and Choi, 2017; Pham et al., 2018). Phytochromes suppress hypocotyl negative gravitropism by inhibiting PIFs in the endodermis (Shin et al., 2009; Kim et al., 2011). In the dark, Arabidopsis seedlings possess endodermal amyloplasts with large starch granules that act as statoliths. These allow them to grow against the direction of gravity, thus showing hypocotyl negative gravitropism. In monochromatic red or far-red light, which activate phyB and phyA, respectively, these endodermal plastids show much smaller starch granules. This causes the seedlings to grow in random directions, displaying hypocotyl agravitropism. Unlike wild type seedlings, *pifQ* mutant seedlings (lacking *PIF1, PIF3, PIF4*, and *PIF5*) have endodermal plastids with small starch granules and show reduced hypocotyl negative gravitropism in the dark (Shin et al., 2009; Kim et al., 2011). These *pifQ* mutant phenotypes are fully rescued by endodermis-specific expression of *PIF1*. This indicates PIFs promote hypocotyl negative gravitropism in the endodermis in part by maintaining large starch granules in endodermal amyloplasts in the dark (Kim et al., 2011). It is not yet clear how PIFs promote starch granule formation and hypocotyl negative gravitropism in the endodermis.

In a mutagenesis screen with *SCRp:PIF1/pifQ*, a transgenic line expressing *PIF1* under the endodermis-specific *SCARECROW* (*SCR*) promoter in the *pifQ* mutant background, we isolated several *reduced gravitropic* (*rgv*) mutants. We then further characterized *rgv1*, allelic to *ESV1*, mutants, which lack starch granules both in the endodermis and the root columella in the dark. By analyzing Arabidopsis and rice *ESV1* mutants as well as transgenic plants expressing *ESV1* from liverwort, Arabidopsis, and rice, we have identified *ESV1* as a functionally conserved protein that promotes gravitropism in land plants via its role in the formation of starch granules.

## Results

### Three *reduced gravitropic* Mutants Lack Starch Granules in the Endodermis

**Figure 1:**
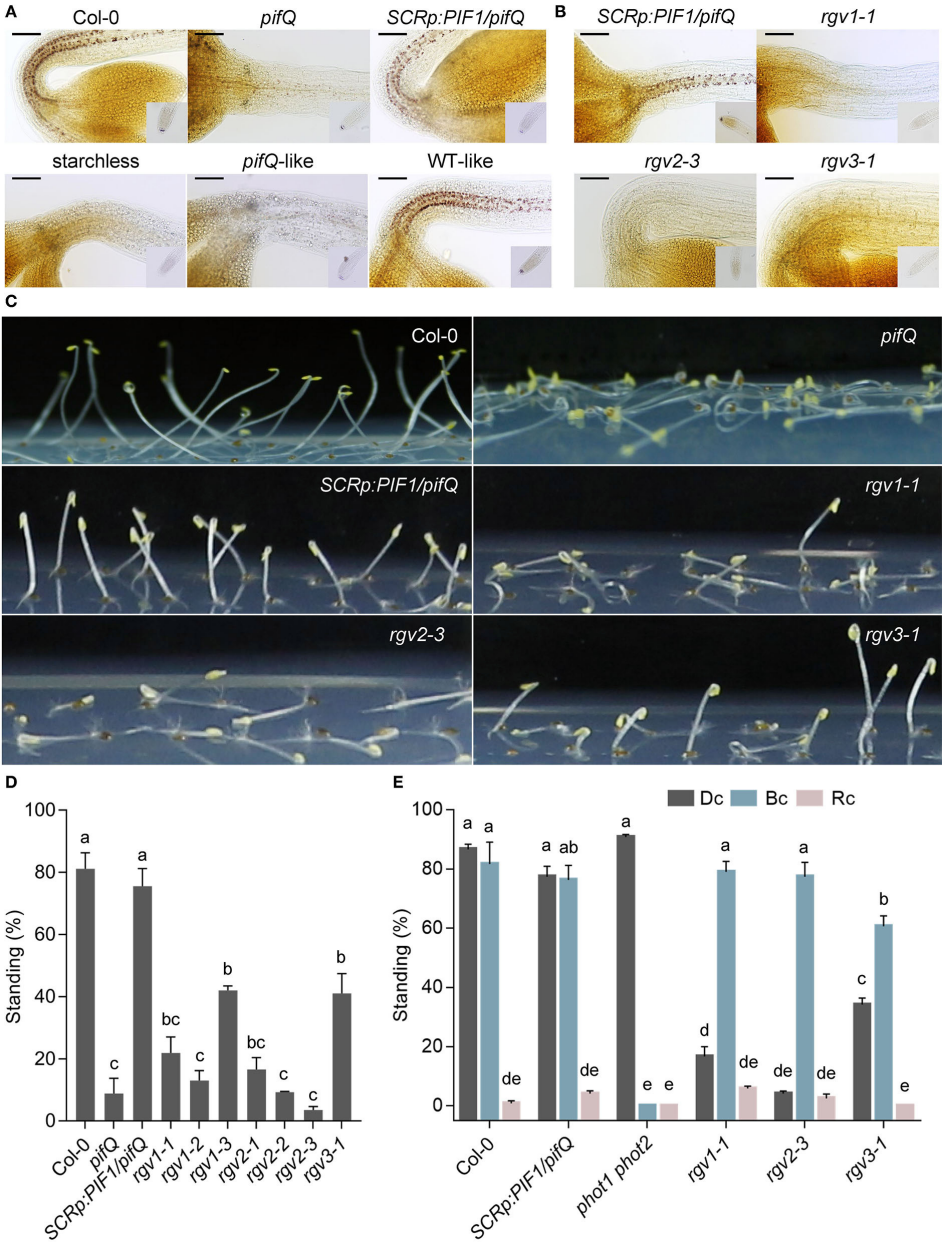
Three *reduced gravitropic* (*rgv*) mutants lack endodermal starch granules and show reduced hypocotyl negative gravitropism. **(A)** Classification of *rgv* mutants into three groups based on the presence of endodermal starch granules. Endodermal starch granules were visualized by staining 3-day-old dark-grown seedlings with Lugol's iodine. Col-0: wild type, *pifQ*: *pif1 pif3 pif4 pif5* mutant, *SCRp:PIF1/pifQ*: the parental line expressing *PIF1* under the *SCR* promoter in the *pifQ* mutant background, starchless: *rgv* mutants with no endodermal starch granules, *pifQ*-like: *rgv* mutants with reduced starch granules, WT-like: *rgv* mutants with clear endodermal starch granules. Scale bar = 100 μm. **(B)** Three complementation groups of starchless *rgv* mutants. *rgv1-1, rgv2-3*, and *rgv3-1* represent each of the three starchless complementation groups. Scale bar = 100 μm. **(C)** Reduced hypocotyl negative gravitropism of three starchless *rgv* mutants. For the hypocotyl negative gravitropism assay, seedlings were grown for 3 days in the dark on horizontal plates. Images of three starchless *rgv* mutants are shown. **(D)** Quantification of reduced hypocotyl negative gravitropism for three starchless *rgv* mutants. Seedlings were grown as in **(C)**. The degree of negative gravitropism is presented as the percentage of standing seedlings in the dark. Seedlings with cotyledons not touching the agar surface were counted as standing. Letters indicate statistical significance determined by an ANOVA with Tukey's HSD *post-hoc* test for multiple comparisons (*p* < 0.01). Error bars=SEM (*n* = 3 biological replicates, 40 seedlings each). **(E)** Normal blue light phototropism of three starchless *rgv* mutants. Standing seedlings were counted after being grown for 4 days in different overhead light conditions on horizontal plates. Percentages of standing seedlings in the dark and under red light represent the degrees of hypocotyl negative gravitropism in the dark and under red light, respectively. The percentage of standing seedlings under blue light represent the degree of phototropism. Dc: continuous dark, Rc: continuous red light (20 μmol/m^2^s), Bc: continuous blue light (1 μmol/m^2^s). Letters indicate statistical significance determined by an ANOVA with Tukey's HSD *post-hoc* test for multiple comparisons (*p* < 0.01). Error bars=SEM (*n* = 3 biological replicates, 40 seedlings each).

We previously showed that the endodermis-specific expression of *PIF1* is sufficient to rescue the endodermal starch granule and hypocotyl negative gravitropism phenotypes of *pifQ* mutants in the dark (Kim et al., 2011). To identify the molecular components that regulate hypocotyl negative gravitropism downstream of endodermal PIF1, we performed an ethyl methansulfonate (EMS) mutagenesis screen using a transgenic parental line expressing *PIF1* under the endodermis-specific *SCR* promoter in *pifQ* mutant background (*SCRp:PIF1/pifQ*). From this screen, we isolated 30 *reduced gravitropic* (*rgv*) mutants that show reduced hypocotyl negative gravitropism in the dark. We classified these 30 *rgv* mutants into three groups based on the status of their endodermal starch granules when stained by Lugol's iodine: a starchless group lacking starch granules, a *pifQ*-like group with reduced starch granules, and a WT-like group with large starch granules (Figure 1A). First, we characterized the starchless group of mutants and found that it includes seven mutants that belong to three complementation groups (*rgv1-1* to *rgv1-3, rgv2-1* to *rgv2-2*, and *rgv3-1*). Both *rgv1* and *rgv2* mutants lack starch granules not only in the endodermis but also in the root columella, whereas the *rgv3* mutant lacks starch granules in the endodermis but has weakly stained granules in the root columella (Figure 1B).

All three groups of *rgv* mutants show reduced hypocotyl negative gravitropism. When grown on horizontal agar plates in the dark, the three types of *rgv* mutants, like the *pifQ* mutant, grow along on the agar surface instead of upward like wild type and parental seedlings (Figures 1C,D). The three *rgv* mutants also show reduced bending toward the new direction of gravity when dark-grown seedlings are rotated by 135 degrees (Supplementary Figure 1A). Reduced gravitropic responses of these *rgv* mutants are not due to either a growth defect or an inherent defect in asymmetric growth of their hypocotyls; We found hypocotyl lengths of all three *rgv* mutants are not much different from that of parental line (Supplementary Figure 2). We also found all three *rgv* mutants either grow upward in the presence of overhead blue light illumination (Figure 1E) or bend toward blue light illuminated from the side (Supplementary Figure 1B). In contrast, the *PHOTOTROPIN* double mutant, *phot1 phot2*, does not bend toward blue light. Thus, the three *rgv* mutants show proper phototropism, which requires asymmetric growth. In addition, adult *rgv* mutants produce axillary branches with wider branch angles (Supplementary Figure 3). Together, these results indicate that these three *rgv* mutants lack endodermal starch granules and display reduced hypocotyl negative gravitropism.

### Identification of *RGV1, RGV2*, and *RGV3* Genes

We first determined if any of the *rgv* mutants are allelic to known starch biosynthetic mutants. Of these, the *pgm* and *ADP-glucose pyrophosphorylase small subunit 1* (*aps1*) mutants, like *rgv1* and *rgv2*, lack starch granules in both the hypocotyl endodermis and the root columella. The *isoamylase 1* (*isa1*) and *isoamylase 2* (*isa2*) mutants, like *rgv3*, lack starch granules in the hypocotyl endodermis but do show stainable granules in the root columella (Supplementary Figure 4A). We tested allelism by crossing the *rgv* mutants to these known starch biosynthetic mutants and staining the F_1_ progeny with Lugol's iodine. The F_1_ progeny of the cross between *rgv2* and *pgm* did not show any starch granules in either the endodermis or the root columella, indicating *rgv2* is allelic to *pgm* (Supplementary Figure 4B). Similarly, *rgv3* is allelic to *isa1*. The *rgv1* mutant, however, is neither allelic to *pgm*, nor to *aps1*. We further sequenced the genomic locations of *PGM, APS1, ISA1*, and *ISA2* in the *rgv* mutants. Consistent with their apparent allelism, we found the *rgv2* mutants have mutations in the *PGM* gene and the *rgv3* mutant has a mutation in the *ISA1* gene (Supplementary Figure 4C). The *rgv1* mutants, however, did not show any mutation in *PGM, APS1, ISA1*, or *ISA2*. These results indicate the *rgv1* mutants are novel mutants that lack starch granules in both the endodermis and the root columella.

**Figure 2:**
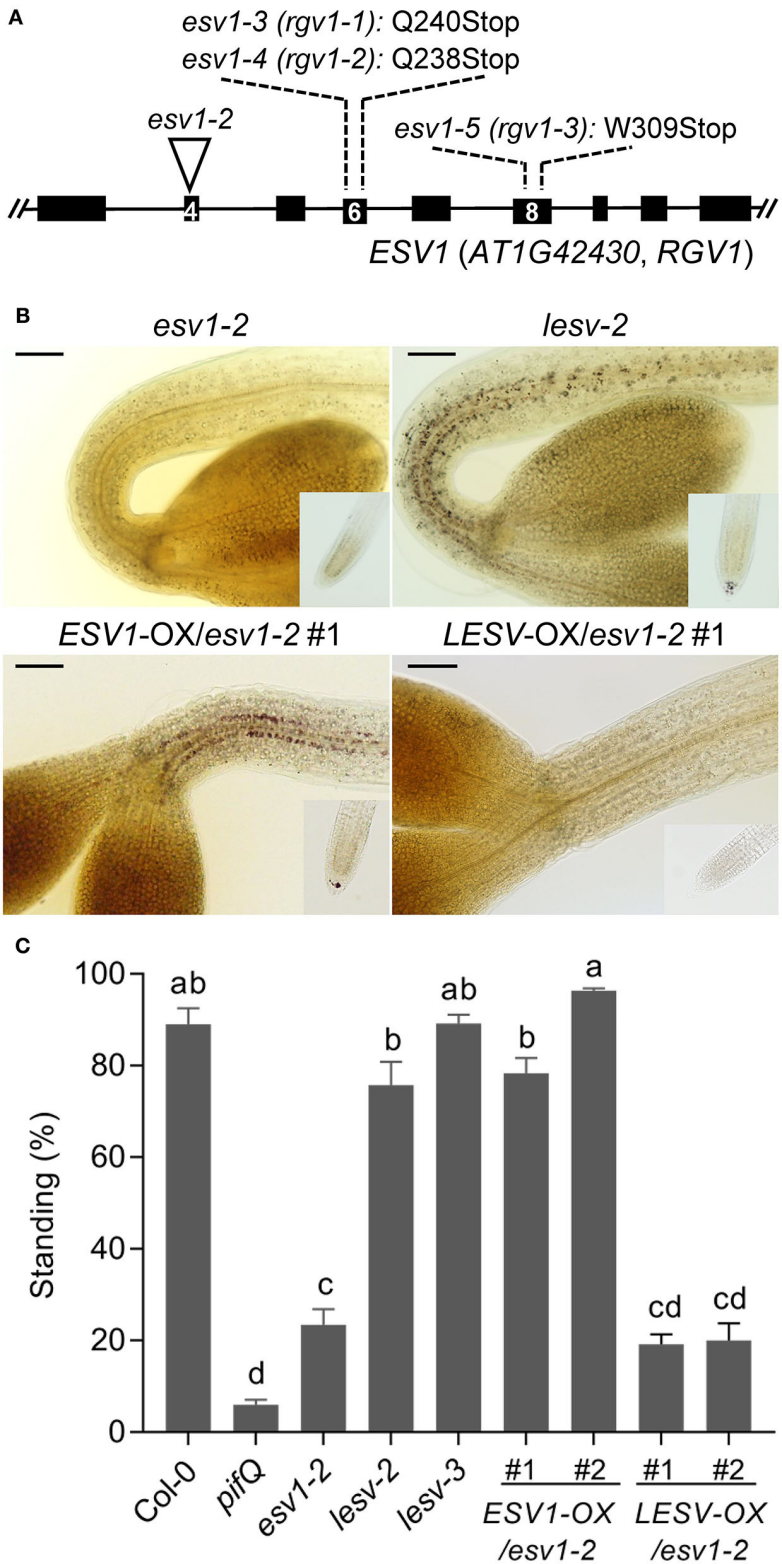
Three *reduced gravitropic 1* mutant alleles have non-sense mutations in *AT1G42430*. **(A)** A diagram showing these three *rgv1* mutant alleles and a T-DNA insertion mutant allele. The mutated loci in these three *rgv1* alleles (*rgv1-1, rgv1-2, rgv1-3*) were determined by whole-genome sequencing. All three *rgv1* alleles have non-sense mutations in *AT1G42430*, indicated by the wild type amino acid with its position number and Stop. A T-DNA insertion line (*ESV1-2, GABI_031C11*) was obtained from the stock center. Black bars: exons, Lines: introns, Numbers in black bars: exon number, Inverted triangle: T-DNA insertion site. **(B)** Restoration of endodermal starch granules in the *ESV1* mutant via ectopic expression of *ESV1* but not *LESV*. Endodermal starch granules were visualized by staining 3-day-old dark-grown seedlings with Lugol's iodine. *ESV1-2*: a T-DNA insertion mutant in *AT1G42430* (*GABI-031C11*), *lesv-2*: a T-DNA insertion mutant in *AT3G55760* (*Salk_006705*), *ESV1*-OX/*ESV1-2*: a transgenic line expressing *ESV1* under the *35S* promoter in the *ESV1-2* mutant background, *LESV*-OX/*ESV1-2*: a transgenic line expressing *LESV* under the *35S* promoter in the *ESV1-2* mutant background. Scale bar = 100 μm. **(C)** Restoration of hypocotyl gravitropism in *ESV1* mutants via the ectopic expression of *ESV1* but not *LESV*. Seedlings were grown for 3 days in the dark on horizontal plates and the degree of negative gravitropism was measured by quantifying the percentage of standing seedlings. Seedlings were counted as standing if their cotyledons did not touch the agar surface. Col-0: wild type, *pifQ*: *pif1 pif3 pif4 pif5* mutant, *ESV1-2*: a T-DNA insertion mutant of *AT1G42430* (*GABI-031C11*), *lesv-2*: a T-DNA insertion mutant of *AT3G55760* (*Salk_006705*), *lesv-3*: a T-DNA insertion mutant of *AT3G55760* (*Salk_006697*), *ESV1*-OX/*ESV1-2*: transgenic plants expressing *ESV1* under the *35S* promoter in the *ESV1-2* mutant background, *LESV*-OX/*ESV1-2*: transgenic plants expressing *LESV* under the *35S* promoter in the *ESV1-2* mutant background. Letters indicate statistical significance determined by an ANOVA with Tukey's HSD *post-hoc* test for multiple comparisons (*p* < 0.01). Error bars=SEM (*n* = 3 biological replicates, 40 seedlings each).

To identify the gene responsible for the *rgv1* phenotype, we sequenced the *rgv1* mutant genomes and found that all three *rgv1* mutants (*rgv1-1, rgv1-2, rgv1-3*) carry non-sense mutations in *AT1G42430*, which is previously reported as *EARLY STARVATION 1* (*ESV1*) (Figure 2A) (Feike et al., 2016). Thus, we will refer the *rgv1* mutant as the *ESV1* mutant hereinafter. To prove that mutations in this gene reduce negative gravitropism, we isolated a T-DNA insertion mutant (*ESV1-2, GABI_031C11*) in this gene. We found that this T-DNA insertion mutant lacks starch granules in both the endodermis and root columella (Figure 2B) and shows reduced hypocotyl negative gravitropism in the dark (Figure 2C). Furthermore, we found that the ectopic expression of *ESV1* under the *35S* promoter in the *ESV1* mutant background (*ESV1*-OX/*ESV1-2*) rescues both endodermal starch granules and hypocotyl negative gravitropism (Figures 2B,C). These results indicate the *rgv1* mutant is caused by mutations in the *ESV1* gene (*AT1G42430*). It is noticeable, however, that the *ESV1* mutant still produces high levels of starch granules in hypocotyl cortex and cotyledons at the end of long day (ZT16), which is different from the *pgm* mutant that does not produce any starch granule and the *phosphoglucoisomerase* (*pgi*) mutant that produces high levels of starch granules in the hypocotyl endodermis but strongly reduced levels in cotyledons at ZT16 (Supplementary Figure 5). At 4 h after the start of night (ZT20), starch granules are depleted in the *ESV1* mutant but not in wild type and the *pgi* mutant. Such starch granule accumulation patterns in different tissues indicate that *ESV1* is essential to form starch granules in the endodermis where starch is formed from transported glucose-6-phosphates and to maintain starch granules from the hasty depletion during the night. Arabidopsis also possesses an *ESV1* homolog (*AT3G55760, LESV*) that is 44% identical at the amino acid level to *ESV1*. Unlike *ESV1* mutants, however, mutations in *LESV* do not disrupt endodermal starch granules (Figure 2B), neither do they affect hypocotyl negative gravitropism in the dark (Figure 2C). The ectopic expression of *LESV* under the *35S* promoter in the *ESV1* mutant background (*LESV*-OX/*ESV1-2*) does not rescue the *ESV1* mutant endodermal starch granule or hypocotyl negative gravitropism phenotypes (Figures 2B,C), indicating *ESV1* and LESV do not have the same function.

*ESV1* is reported to be localized in plastids and prevents the hasty depletion of starch reserves during the night (Feike et al., 2016). Consistent with previous reports, we observed punctate *ESV1*-GFP signal in the chloroplasts of light-grown mesophyll cells (Supplementary Figure 6A) and in the plastids of dark-grown upper hypocotyl endodermal cells possessing large starch granules. In dark-grown hypocotyl epidermal cells that lack starch granules, however, although the *ESV1*-GFP signal is localized to plastids, it does not form punctate signals (Supplementary Figures 6B,C). This is consistent with a previous report that *ESV1* is a plastid-localized protein that can associate with starch granules.

### *ESV1* Acts Cell Autonomously in the Endodermis to Promote Hypocotyl Negative Gravitropism *via* Its Role in Starch Granule Formation

**Figure 3:**
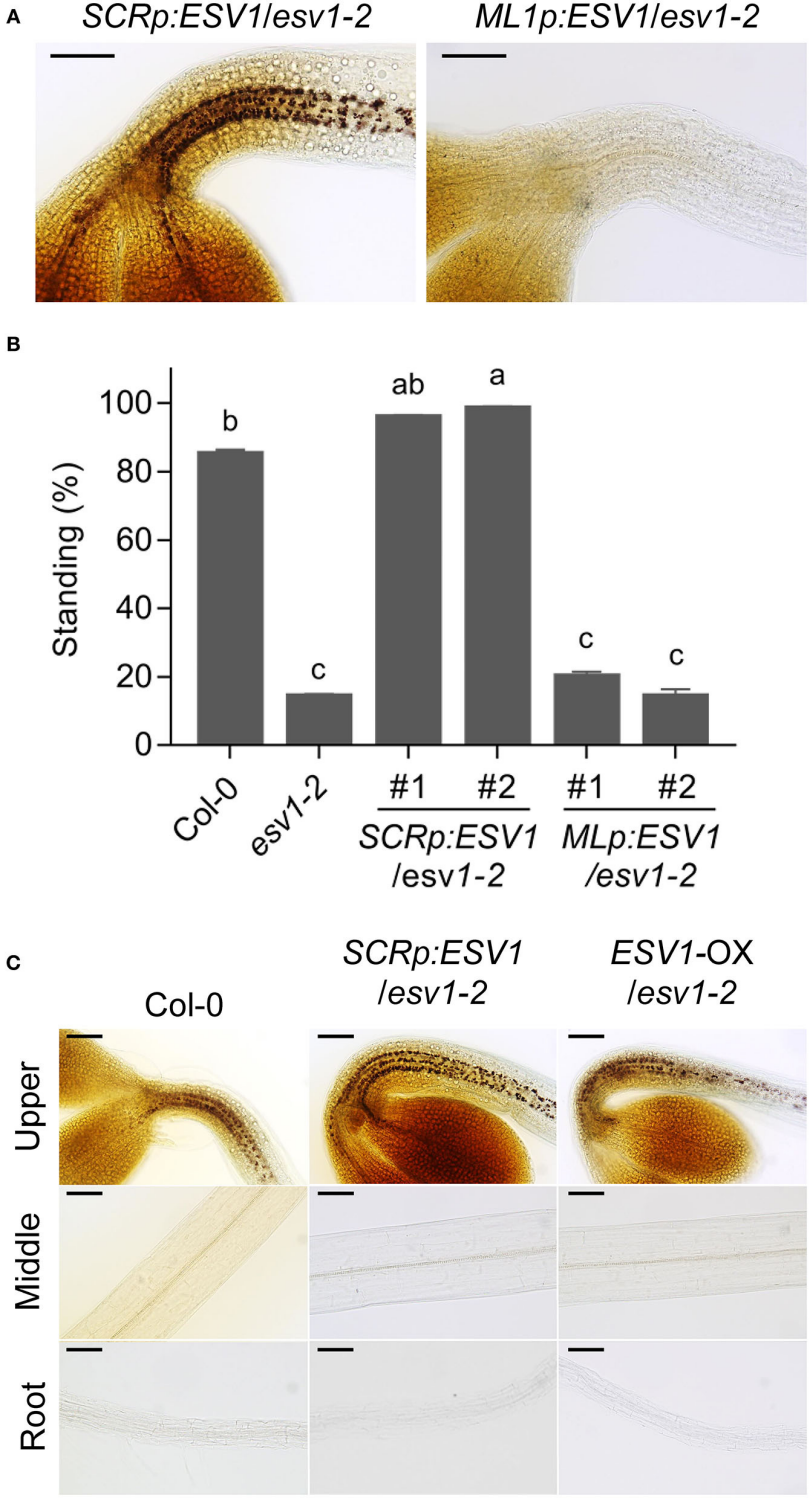
*ESV1* promotes hypocotyl negative gravitropism in the endodermis. **(A)** Restoration of endodermal starch granules by endodermal but not epidermal *ESV1*. Endodermal starch granules were visualized by staining 3-day-old dark-grown transgenic seedlings with Lugol's iodine. *SCRp:ESV1*/*ESV1-2*: transgenic plants expressing *ESV1* under the endodermis-specific *SCR* promoter in the *ESV1-2* mutant background, *ML1p:ESV1*/*ESV1-2*: transgenic plants expressing *ESV1* under the epidermis-specific *ML1* promoter in the *ESV1-2* mutant background. Scale bar = 100 μm. **(B)** Restoration of hypocotyl negative gravitropism by endodermal but not epidermal *ESV1*. Seedlings were grown for 3 days in the dark on horizontal plates taking the percentage of standing seedlings as an indicator of the level of negative gravitropism. Standing seedlings had cotyledons that did not touch the agar surface. Two independent transgenic lines for each tissue-specific promoter are indicated. Letters indicate statistical significance determined by an ANOVA with Tukey's HSD *post-hoc* test for multiple comparisons (*p* < 0.01). Error bars=SEM (*n* = 3 biological replicates, 40 seedlings each). **(C)** Insufficiency of *ESV1* for the ectopic formation of starch granules in the lower hypocotyl and roots. Starch granules were visualized by staining 3-day-old dark-grown transgenic seedlings with Lugol's iodine. *ESV1*-OX/*ESV1-2*: transgenic plant expressing *ESV1* under the *35S* promoter in the *ESV1-2* mutant background. Scale bar = 100 μm.

Since the endodermis is a heterotrophic tissue that obtains carbon from other tissues (José Muñoz et al., 2006), we asked if *ESV1* acts cell-autonomously in the endodermis to assist in the formation of starch granules, subsequently leading to a hypocotyl negative gravitropism phenotype when it is mutated. We generated transgenic lines expressing *ESV1* under the control of the endodermis-specific *SCR* promoter in the *ESV1-2* mutant background (*SCRp:ESV1*/*ESV1-2*) and transgenic lines expressing *ESV1* under the control of the epidermis-specific *MERISTEM LAYER 1* (*ML1*) promoter in the same *ESV1-2* mutant background (*ML1p:ESV1*/*ESV1-2*) (Di Laurenzio et al., 1996; Sessions et al., 1999; Heidstra et al., 2004). When we stained these seedlings with Lugol's iodine, we observed clear starch granules in the upper hypocotyl endodermis of dark-grown *SCRp:ESV1*/*ESV1-2* lines, but not in *ML1p:ESV1*/*ESV1-2* lines (Figure 3A). This indicates that *ESV1* expressed in the endodermis but not the epidermis is able to restore starch granules in the endodermis. Consistent with this restoration of endodermal starch granules, we also observed a rescue of the hypocotyl negative gravitropism phenotype only in the *SCRp:ESV1*/*ESV1-2* lines, not in the *ML1p:ESV1*/*ESV1-2* lines (Figure 3B). The inability of epidermis-specific *ESV1* to rescue starch granules and the hypocotyl negative gravitropism phenotype was not due to lower levels of *ESV1* mRNA expression; *ML1p:ESV1*/*ESV1-2* line expresses similar levels of *ESV1* mRNA than *SCRp:ESV1*/*ESV1-2* line (Supplementary Figure 7). Together, these results indicate *ESV1* acts cell autonomously in the endodermis in the formation of starch granules, subsequently contributing to hypocotyl negative gravitropism.

We found, however, that the ectopic expression of *ESV1* is itself insufficient to induce the production of starch granules. The *SCR* promoter is active not only in the hypocotyl endodermis but also in the endodermis of other tissues including roots. The *SCRp:ESV1*/*ESV1-2* line, however, forms starch granules only in the upper hypocotyl endodermis, not in the middle hypocotyl endodermis or root endodermis (Figure 3C). Neither does the expression of *ESV1* under the control of the *ML1* promoter induce the formation of starch granules in the epidermis (Figure 3A). Even when we expressed *ESV1* ubiquitously under the control of the *35S* promoter (*ESV1-*OX*/ESV1-2*), we only observed starch granule formation in the upper hypocotyl endodermis (Figure 3C). These results indicate *ESV1* is necessary but insufficient for ectopic starch granule formation.

### Phytochromes Do Not Remove Starch Granules by Repressing *ESV1* Expression

**Figure 4:**
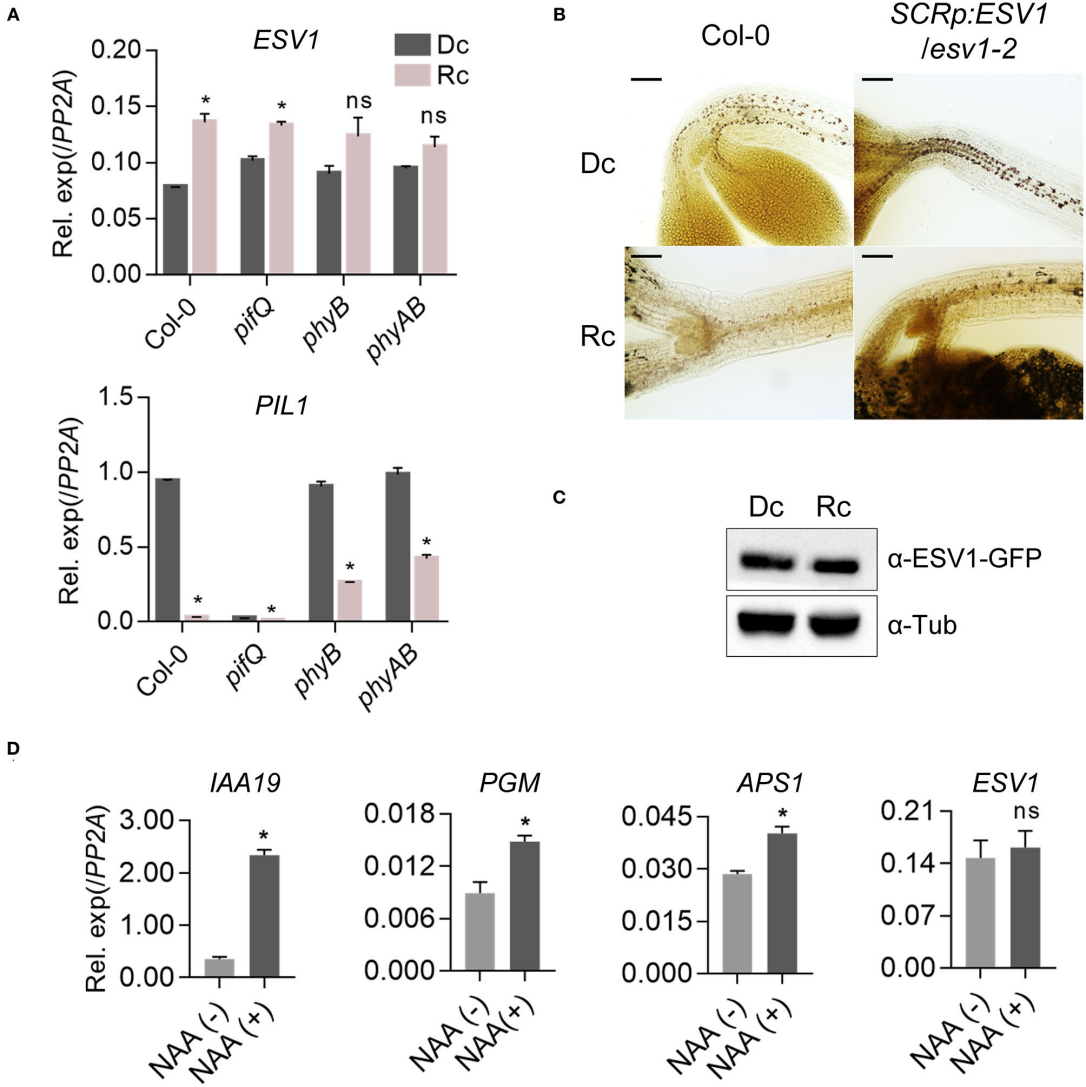
PhyB does not repress *ESV1* to degrade endodermal starch granules. **(A)** No repression of *ESV1* mRNA expression by red light. Three-day-old seedlings grown either in the dark (Dc) or red light (Rc) were used for mRNA expression analysis. Col-0: wild type, *pifQ*: *pif1 pif3 pif4 pif5* mutant. *phyB*: *phyB-9* mutant. *phyAB*: *phyA-211 phyB-9* double mutant. Relative mRNA expression was normalized by *PP2A*. Asterisks indicate significant differences from dark condition (*p < 0.05; Student's *t*-test). n.s indicates no significant difference. Error bars=SEM (*n* = 3 biological replicates). **(B)** Degradation of starch granules by red light even in transgenic seedlings expressing *ESV1* under the *SCR* promoter. Endodermal starch granules were visualized by staining 3-day-old seedlings grown in dark (Dc) or red light (Rc) with Lugol's iodine. Col-0: wild type, *SCRp:ESV1*/*ESV1-2*: transgenic plants expressing *ESV1* by endodermis-specific *SCR* promoter in the *ESV1-2* mutant background. Scale bar = 100 μm. **(C)** No destabilization of *ESV1* protein by red light. Transgenic seedlings expressing *ESV1-GFP* under the *35S* promoter in the wild type background (*35Sp:ESV1-GFP*) were grown for 4 days either in the dark (Dc) or under red light (Rc) and *ESV1*-GFP protein levels were detected by Western blot using a GFP-specific antibody (α-GFP). α-tubulin was detected using an α-tubulin-specific antibody (α-Tub). The intensity of *ESV1*-GFP was normalized by α-tublin (*n* = 3, biological replicates). **(D)** No activation of *ESV1* mRNA expression by auxin. Seven-day-old seedlings grown in the light on a nylon mesh were transferred to media with [NAA(+)] or without [NAA(-)] auxin. At 48 h after the incubation, the root tips were removed with a blade and collected for RNA purification. Relative mRNA expression was normalized to *PP2A*. Asterisks indicate significant differences from NAA(-) condition (*p < 0.05; Student's *t*-test). ns indicates no significant difference. Error bars=SEM (*n* = 3 biological replicates).

PIFs promote hypocotyl negative gravitropism partly by maintaining starch granules in the endodermis in the dark. phyB suppresses hypocotyl negative gravitropism by inhibiting PIFs in red light (Kim et al., 2011). Since *ESV1* is necessary to maintain starch granules in the endodermis, we asked if phyB and PIFs regulate starch granules through *ESV1*. We first determined the expression of *ESV1* mRNA. In contrast to the expression of *PHYTOCHROME INTERACTING FACTOR 3-LIKE 1* (*PIL1*), a PIF direct target gene used as a marker (Park et al., 2018), we found no evidence of red light-induced *ESV1* mRNA repression in wild type seedlings or de-repression in *phyB* mutant seedlings (Figure 4A). This indicates phyB does not repress the expression of *ESV1* mRNA in red light. We also found no evidence of *ESV1* mRNA repression in *pifQ* mutants in the dark, confirming that PIFs do not activate the expression of *ESV1* mRNA in the dark. Furthermore, red light still reduces endodermal starch granules in *SCRp:ESV1*/*ESV1-2* seedlings (Figure 4B) expressing *ESV1* mRNA under the control of the *SCR* promoter. These results support that phyB and PIFs do not regulate starch granules via their regulation of *ESV1* mRNA expression. We next used a transgenic line expressing *ESV1*-GFP under the control of the 35S promoter to determine whether phyB reduces starch granules by destabilizing *ESV1* protein. We found that red light does not reduce *ESV1* protein levels (Figure 4C). Neither does red light reduce polysome-bound *ESV1* mRNA levels (Liu et al., 2013), suggesting phytochromes do not repress the translation of *ESV1* mRNA in red light. Together, these results indicate phyB removes endodermal starch granules in red light neither by repressing *ESV1* mRNA levels nor by decreasing *ESV1* protein levels. Auxin is known to promote starch granule accumulation in root tips by activating the expression of starch biosynthetic genes, including *PGM* and *APS1* (Zhang et al., 2019a). Auxin does not, however, regulate the expression of *ESV1* mRNA (Figure 4D). Together, our results indicate *ESV1* mRNA expression is not the main point of regulation in the control of starch granule accumulation.

### *ESV1* Gene From a Liverwort or Rice Can Complement Arabidopsis *ESV1* Mutant

**Figure 5:**
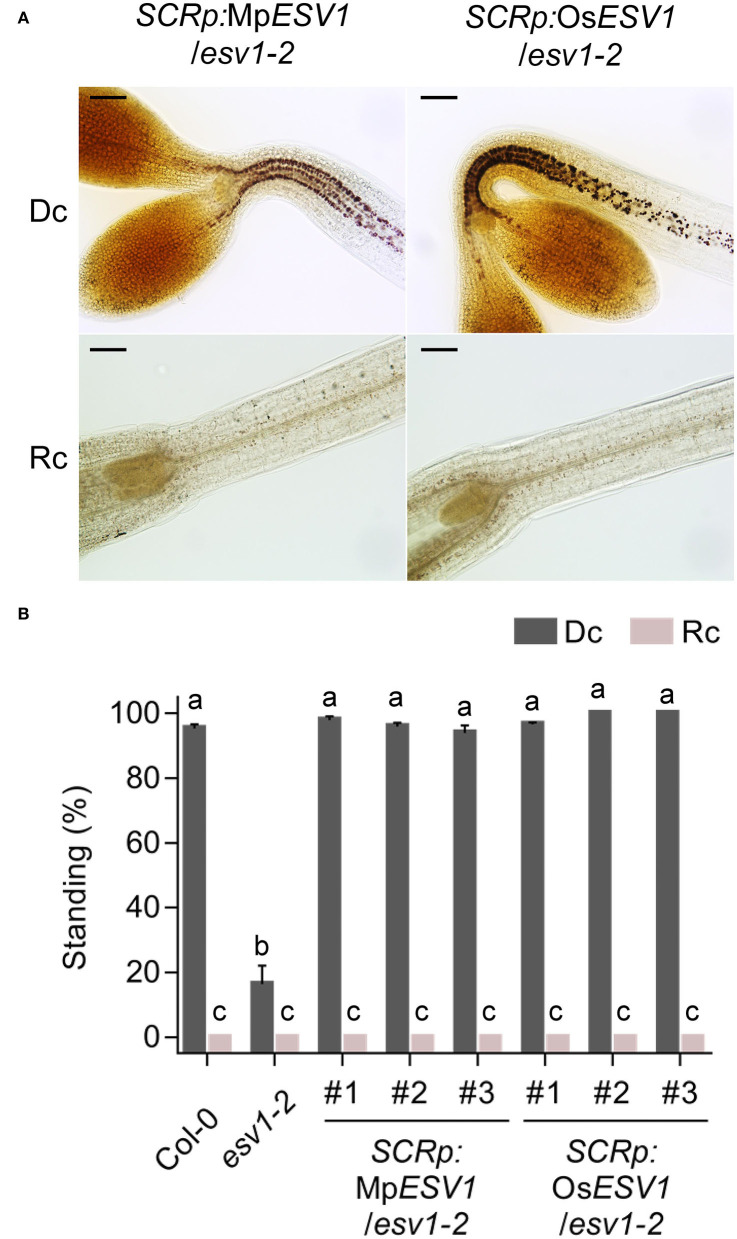
Liverwort and rice *ESV1* genes can rescue the Arabidopsis *ESV1* mutant. **(A)** Liverwort and rice *ESV1* rescue endodermal starch granules in the Arabidopsis *ESV1* mutant background. Transgenic seedlings expressing either liverwort or rice *ESV1* under the *SCR* promoter in the *ESV1-2* mutant background (*SCRp:*Mp*ESV1*/*ESV1-2, SCRp:*Os*ESV1*/*ESV1-2*, were grown for 3 days either in the dark (Dc) or in red light (Rc). Then, starch granules were visualized by staining the seedlings with Lugol's iodine. Scale bar = 100 μm. **(B)** Liverwort and rice *ESV1* rescue hypocotyl negative gravitropism in the Arabidopsis *ESV1* mutant background. Seedlings were grown for 3 days in the dark (Dc) or in red light (Rc) on horizontal plates. The percentage of standing seedlings indicates the degree of negative gravitropism. Seedlings whose cotyledons did not touch the agar surface were counted as standing. The numbers indicate independent transgenic lines. Letters indicate statistical significance determined by an ANOVA with Tukey's HSD *post-hoc* test for multiple comparisons (*p* < 0.01). Error bars=SEM (*n* = 3 biological replicates, 40 seedlings each).

*ESV1* orthologs are present in the genomes of various starch-producing eukaryotic green lineages including liverwort (*Marchantia polymorpha*) and rice (*Oryza sativa)* but not in those of cyanobacteria that do not produce starch. To investigate the relationship between *ESV1*'s conservation and the formation of starch granules, we generated transgenic Arabidopsis seedlings expressing either liverwort *ESV1* (*Marchantia polymorpha*, Mp-*ESV1* (*Mapoly0189s0007*), 74% amino acid identity to At-*ESV1*) or rice *ESV1* (*Oryza sativa*, Os-*ESV1*(*LOC_Os03g04100*), 88% amino acid identity to At-*ESV1*) under the control of the endodermis-specific *SCR* promoter in the *ESV1* mutant background (*SCRp:*Mp*ESV1/ESV1-2, SCRp:*Os*ESV1/ESV1-2*). If the *ESV1* proteins from these species are functionally conserved, these transgenic seedlings should produce endodermal starch granules. We found that both Mp-*ESV1* and Os-*ESV1* fully rescue the production of endodermal starch granules (Figure 5A) and hypocotyl negative gravitropism in Arabidopsis *ESV1* mutant seedlings (Figure 5B). Furthermore, in *SCRp*:Mp*ESV1*/*ESV1-2* and *SCRp*:Os*ESV1*/*ESV1-2* seedlings, just as in *SCRp*:*ESV1*/*ESV1-2* seedlings, red light inhibits the production of starch granules and hypocotyl negative gravitropism (Figures 5A,B). Together, these results indicate *ESV1* proteins from liverwort and rice are functionally conserved in Arabidopsis.

### Rice *ESV1* Promotes Tight Packing of Starch Granules in Grains

**Figure 6:**
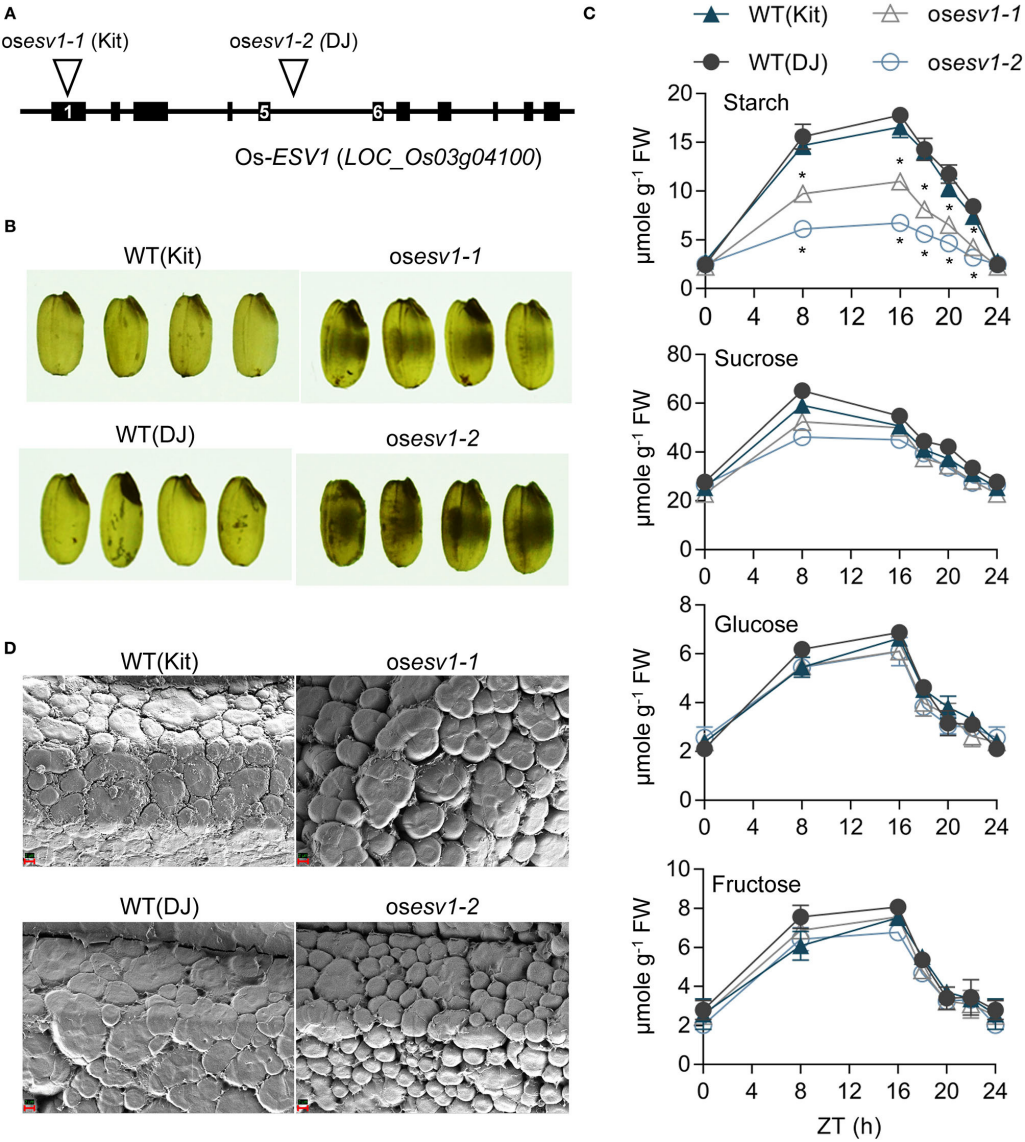
Rice *ESV1* promotes the accumulation of starch in leaves and the tight packing of starch granules in grains. **(A)** A diagram showing two rice *ESV1* mutant alleles. Two rice T-DNA insertion lines (os*ESV1-1*, os*ESV1-2*) were isolated from the rice stock center. Kitaake (Kit) and Dongjin (DJ) in parenthesis are their respective wild type cultivars. Back bars: exons, Lines: introns, numbers in black bars: exon numbers, inverted triangle: T-DNA insertion locus. **(B)** Chalky grains of rice *ESV1* mutants. Grain images were taken on a white light box. Black patches in the grains indicate chalky areas. **(C)** Reduced levels of starch but not soluble sugars in rice *ESV1* mutant leaves. Leaves of 6-week-old rice plants grown under a 14/10 light/dark cycle in a greenhouse were sampled at each time point and the levels of glucose, fructose, sucrose, and starch were determined. Asterisks indicate significant differences from the respective wild type (*p < 0.05; Student's *t*-test) (Supplementary Table 1). Error bars=SEM (*n* = 3 biological replicates). **(D)** SEM images showing loosely packed starch granules of fractured *ESV1* mutant rice grains. Red scale bars appear in the lower left corners (2 μm).

The fact that Mp-*ESV1* and Os-*ESV1* rescue Arabidopsis *ESV1* mutant phenotypes suggests *ESV1* is a functionally conserved protein that promotes gravitropism via its role in starch granule formation in Arabidopsis and in other plants. We therefore investigated the function of rice *ESV1* by characterizing two independent rice T-DNA insertion mutants (os*ESV1-1*, os*ESV1-2*) in two different rice cultivars (Figure 6A). These two rice *ESV1* mutants have no obvious growth defects and bear starch-filled grains in greenhouse conditions under a 14-h-light/10-h-dark photoperiod (Figure 6B). This is in contrast with the rice starch biosynthetic mutants *ADP-glucose pyrophosphorylase small subunit 2* (os*aps2*) and *ADP-glucose pyrophosphorylase large subunit 2* (os*apl2*) mutants, which produce shrunken grains due to a lack of starch biosynthesis in the endosperm (Lee et al., 2007), and the os*pgm* and *ADP-glucose pyrophosphorylase large subunit 4* (os*apl4*) mutants, which are male sterile because of a lack of starch reserves in their pollen grains (Lee et al., 2016). These phenotypic differences suggest rice *ESV1* mutants store enough starch to support grain filling and pollen survival.

In a more detailed analysis, however, we found *ESV1* promotes starch granule formation also in rice (Supplementary Table 1). First, in a quantitative carbohydrate analysis, we found the two rice *ESV1* mutants accumulate lower levels of leaf starch than wild type during the day but deplete starch more slowly at night, resulting in similarly low starch levels at dawn (Figure 6C). Unlike with starch, however, rice *ESV1* mutants accumulate similar levels of leaf glucose, fructose, and sucrose during the day and deplete them similarly at night, indicating *ESV1* is necessary in leaves specifically for high accumulation of starch during the day. Second, we noticed *ESV1* mutant grains are chalky rather than translucent. Since chalky grains are often caused by altered starch granule structure, we examined these structures in high magnification scanning electron microscope images of fractured rice grains. We found that wild type grains have large, tightly packed starch granules with narrow crevices between them, whereas *ESV1* mutant grains have small, loosely packed starch granules with conspicuous crevices between them (Figure 6D). It is likely the crevices between the granules that lead to the chalky appearance of *ESV1* mutant grains as air cavities between the starch granules reflect and refract more light in random directions. Together, these results indicate *ESV1* is necessary in rice for the accumulation of starch in leaves and to form tightly packed starch granules in rice grains.

### *ESV1* Promotes Root Gravitropism *via* Its Role in Starch Granule Formation in the Root Columella of Arabidopsis and Rice

**Figure 7:**
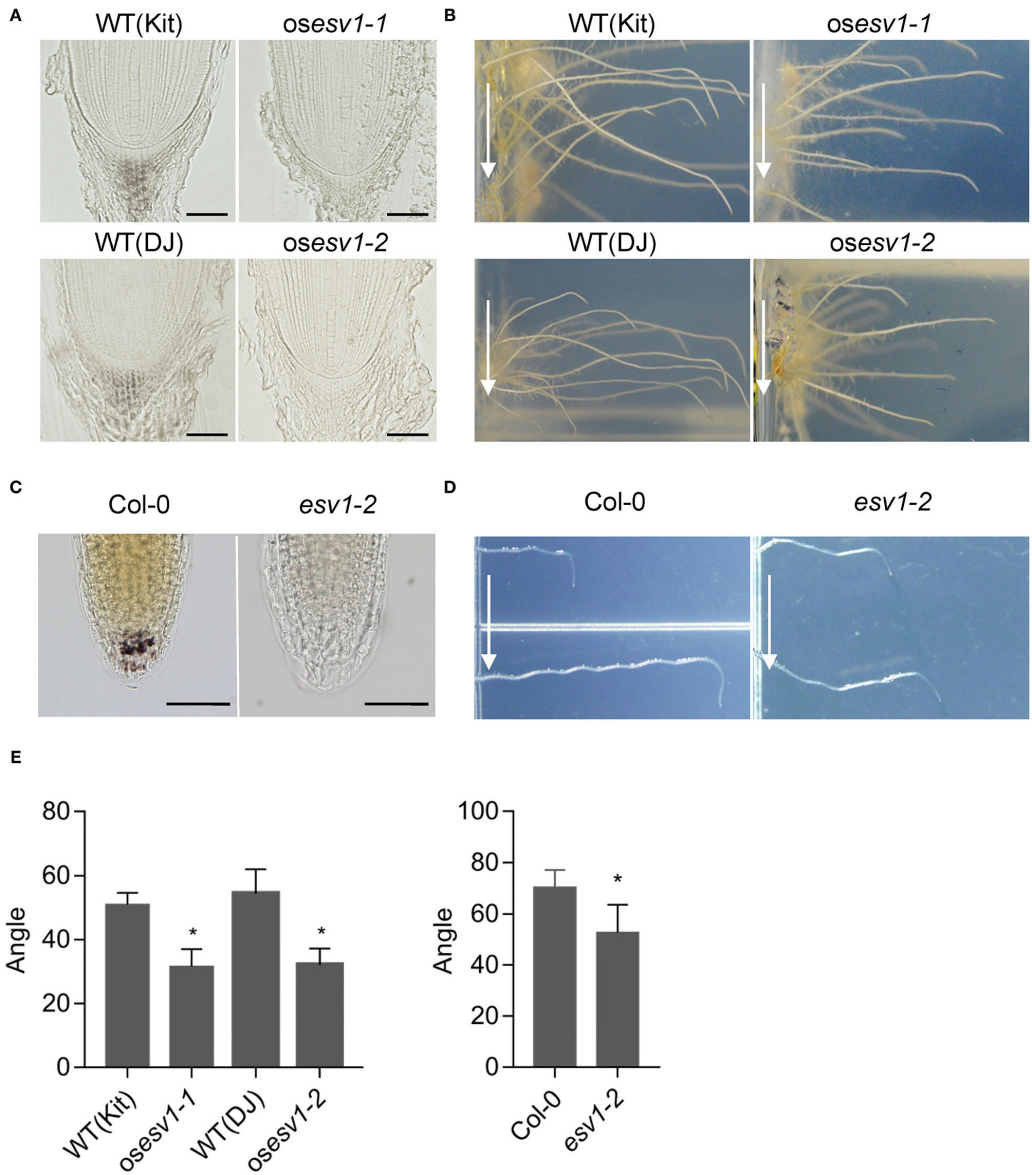
*ESV1* promotes root gravitropism by forming root columella starch granules both in Arabidopsis and rice. **(A)** Absence of root columella starch granules in rice *ESV1* mutants. Root tips were embedded in resin and starch granules were visualized by staining with Lugol's iodine. DJ (Dongjin) and Kit (Kitaake) in parenthesis indicate the respective rice cultivars. Scale bar = 50 μm. **(B)** Reduced root gravitropic responses of rice *ESV1* mutants. Rice seedlings grown on MS-agar for 5 days in the dark were rotated by 90 degrees and grown one more day before taking images to measure bending angles. Arrows indicate the direction of gravity after the rotation. The degree of root gravitropism is presented as the degree of root bending. **(C)** Absence of root columella starch granules in Arabidopsis *ESV1* mutant. Root columella starch granules were visualized by staining 3-day-old dark-grown seedlings with Lugol's iodine. Scale bar = 50 μm. **(D)** Reduced root gravitropic response in Arabidopsis *ESV1* mutants. Arabidopsis seedlings grown on MS-agar for 5 days in the dark were rotated by 90 degrees and grown 12 more hours before taking images to measure the angle of bending. Arrows indicate the direction of gravity after the rotation. The degree of root gravitropism is presented as the degree of root bending. **(E)** Quantitation of root gravitropic responses in rice and Arabidopsis. Seedlings were grown as in **(B,D)**. Asterisks indicate significant differences from wild type (*p < 0.05; Student's *t*-test). Error bars=SD (*n* = 10 for Rice, *n* = 100 for Arabidopsis).

Since Arabidopsis *ESV1* mutants lack starch granules also in root columella cells, we asked whether rice *ESV1* mutants show this same phenotype. After cross-sectioning and staining root tips with Lugol's iodine, we found no starch granules in the root columella cells of rice *ESV1* mutants, whereas the roots of wild type seedlings are filled with starch granules and so are darkly stained (Figure 7A). Starch granules in root columella cells act as statoliths for root gravitropism. To determine whether rice *ESV1* mutant roots show such gravitropism, we reoriented rice seedlings by 90 degrees and measured the bending of their root tips after 24 h of growth in the dark. While the root tips of wild type seedlings bend by more than 50 degrees by 24 h after the reorientation, the roots of rice *ESV1* mutants bend downward by only about 30 degrees (Figures 7B,E). As with rice *ESV1* mutants, Arabidopsis *ESV1* mutants lack starch granules in their root columella cells (Figure 7C) and bend more slowly than wild type roots after a 90-degree reorientation (Figures 7D,E). The rice *ESV1* mutant also show reduced shoot gravitropism (Supplementary Figure 8). Together, these results indicate *ESV1* promotes gravitropism via its role in the formation of starch granules both in Arabidopsis and rice.

### Endodermal Plastids Do Not Sediment in the *ESV1* Mutant

**Figure 8:**
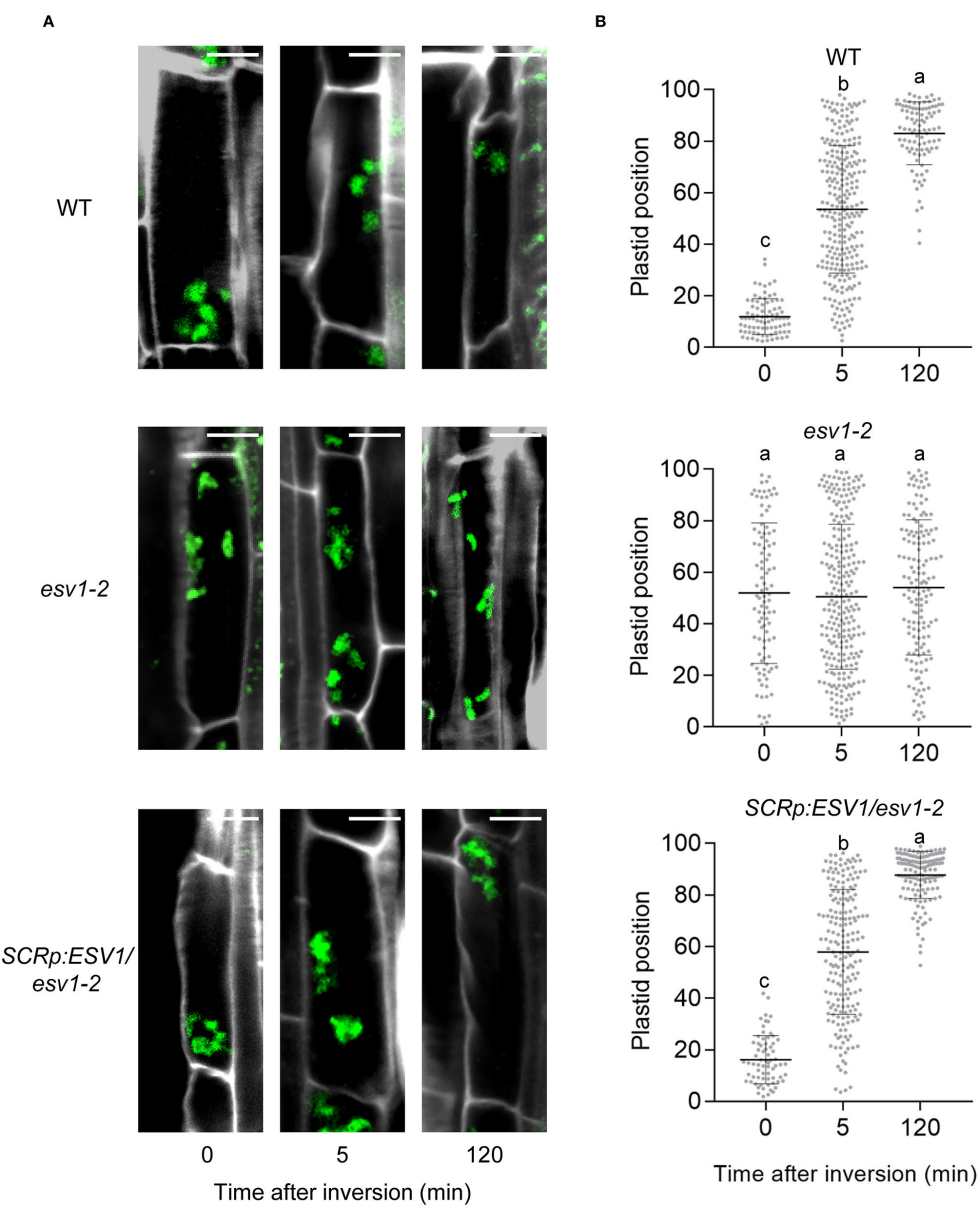
Endodermal plastids of the *ESV1* mutant do not sediment toward the direction of gravity. **(A)** Confocal images showing no sedimentation of endodermal plastids in the *ESV1* mutant. Three day-old dark grown seedlings expressing mCitrine fused with rubisco small subunit transit peptide were inverted in the dark. At the indicated times after inversion, the seedlings were fixed with paraformaldehyde and confocal images were taken after staining their cell walls with Calcofluor white. Cell images were arranged in a typical apical-basal orientation despite the inversion of the seedlings. Green spots indicate endodermal plastids and white lines indicate cell walls. mCitrine fused with a transit peptide was expressed in wild type (WT), the *ESV1* mutant (*ESV1*), and the rescue line (*SCRp:ESV1/ESV1*) background. Scale bar = 10 μm. **(B)** Quantitation showing no sedimentation of endodermal plastids in the *ESV1* mutant. The relative position of each endodermal plastid was calculated by assigning the initial bottom of a cell to 0 and the top of the cell to 100. Each dot indicates one endodermal plastid (*n* > 70). Bars indicate the average and standard deviation. Letters indicate statistical significance determined by an ANOVA with Tukey's HSD *post-hoc* test for multiple comparisons (*p* < 0.01).

We next asked whether the reduced gravitropism of the *ESV1* mutant is due to an inability of its plastids to sediment in the direction of gravity. Amyloplast sedimentation can be tracked with Lugol's iodine staining, but because endodermal plastids in the *ESV1* mutant are not stained by Lugol's iodine, we generated transgenic lines expressing mCitrine fused with a chloroplast transit peptide under the control of the *SCR* promoter. This allowed us to track endodermal plastid sedimentation with a fluorescence microscope (Saito et al., 2005). We grew groups of seedlings on vertical plates in the dark for 3 days and determined the relative positions of the endodermal plastids within their cells either before or after inverting the growth plates. In wild type and *SCRp:ESV1/ESV1-2* seedlings, we found most of the endodermal plastids located at the bottom of the endodermal cells before the inversion, whereas the endodermal plastids in *ESV1-2* mutant seedlings were dispersed throughout the cells (Figures 8A,B). After the inversion, the endodermal plastids in the wild type seedlings began to move toward the new bottoms of the cells. They were dispersed in the cells at 5 min post-inversion and had moved to the new bottoms by 120 min post-inversion (Figures 8A,B). We observed a similar sedimentation pattern in *SCRp*:*ESV1/ESV1-2* seedlings. In the *ESV1-2* mutant seedlings, however, we found the endodermal plastids were dispersed widely regardless of gravity and their inversion status. These results indicate that *ESV1* mutant seedlings display reduced hypocotyl negative gravitropism due to an inability of their endodermal plastids to sediment in the direction of gravity.

## Discussion

### *ESV1* Promotes Gravitropic Responses by Forming Starch Granules in Land Plants

Endodermis-specific expression of *PIF1* restores amyloplasts and hypocotyl negative gravitropism in *pifQ* mutant seedlings (*SCRp:PIF1*/*pifQ*) (Kim et al., 2011). In this study, we mutagenized the *SCRp:PIF1*/*pifQ* line and isolated 30 *reduced gravitropic* (*rgv*) mutants (Figure 1). Among them, we characterized seven mutants lacking starch granules in both the hypocotyl endodermis and root columella. We determined that these seven starchless mutants belong to three complementation groups (*rgv1, rgv2, rgv3*). Of these, *rgv2* and *rgv3* are allelic to the *pgm* and *isa1* mutants, respectively (Supplementary Figure 4C). Using whole genome sequencing, we identified in three *rgv1* alleles point mutations in *AT1G42430*, which is also known as *EARLY STARVATION 1* (*ESV1*) (Figure 2A). *ESV1* encodes a plastid-localized tryptophan-rich protein that has been suggested to prevent hasty depletion of starch overnight either by altering starch matrix organization or by controlling glucan phosphorylation via dikinase (Feike et al., 2016; Malinova et al., 2018). We show *ESV1* acts cell-autonomously in the endodermis to promote hypocotyl negative gravitropism through its role in starch granule formation (Figures 3A,B). *ESV1* homologs are found in starch-accumulating green algae and land plants. We found that *ESV1* orthologs from the liverwort *Marchantia polymorpha* and the monocot rice plant *Oryza sativa* can rescue endodermal starch granules and hypocotyl negative gravitropism in the Arabidopsis *ESV1* mutant (Figure 5). This suggests the function of *ESV1* is conserved. Furthermore, the grains of rice *ESV1* mutants are filled with loosely packed starch granules (Figure 6D), and the root columella produce no starch granules at all (Figure 7A). This loss of starch granules in the root columella reduces root gravitropism both in Arabidopsis and rice (Figures 7B,D). Together, our results indicate the function of *ESV1* in promoting gravitropic responses via its role in starch granule formation has been conserved in plants.

It should be noted, however, that not all green plant lineages that possess *ESV1* homologs use starch-filled amyloplasts as gravity sensors. Genome sequence information indicates that *ESV1* homologs are present not only in starch-producing land plants including liverwort, rice, and Arabidopsis, but also in starch-producing unicellular green algae including *Chlamydomonas* and starch-producing multicellular green algae including *Chara*. Land plants sense gravity using starch-filled amyloplasts as gravity sensors and bend toward or against the direction of gravity, displaying either positive or negative gravitropism (Zhang et al., 2019b). Unicellular *Chlamydomonas* also senses gravity and swims against the direction of gravity, displaying negative gravitaxis (Bean, 1977; Kam et al., 1999). Unlike land plants, however, *Chlamydomonas* possesses a single cup-shaped chloroplast and does not develop starch-filled amyloplasts. Instead, starch granules within the chloroplast encapsulate a matrix containing Rubisco and form a sub-organellar compartment called a pyrenoid (Ramazanov et al., 1994; Giordano et al., 2005). The exact mechanism by which *Chlamydomonas* senses gravity remains poorly understood, but it has been suggested that it is the whole body that senses gravity rather than just the starch-covered pyrenoid (Roberts, 2006). Similarly, the multicellular green algae *Chara* synthesizes starch granules but senses gravity using barium sulfate vesicles for its rhizoid gravitropism rather than starch-filled amyloplasts (Limbach et al., 2005). In contrast to green algae, the protonemata or caulonemata of bryophytes (early land plants), which include *Ceratodon, Funaria*, and *Physcomitrella*, possess sedimentable starch-filled amyloplasts in their growing tips and display negative gravitropism (Jenkins et al., 1986; Walker and Sack, 1990; Schwuchow et al., 1995). This presence of starch granule-dependent gravity sensing in bryophytes but not in green algae suggests the promotion of gravitropic responses by *ESV1* via its role in starch granule formation is likely a derived feature in land plants.

### *ESV1* Is Insufficient for Ectopic Starch Granule Production

Although *ESV1* is necessary for the formation of starch granules in the endodermis and root columella, *ESV1* is neither necessary nor sufficient to induce starch granule formation in other tissues and organs. Both Arabidopsis and rice *ESV1* mutants still accumulate starch granules in leaves during the day, albeit at reduced levels (Figure 6C) (Feike et al., 2016). Redundant activity of homologous genes is unlikely responsible for the formation of these residual starch granules in leaves because none of the homologous genes complement the *ESV1* mutant. This suggests these homologous proteins do not have redundant functions with *ESV1* and further confirms *ESV1* is inessential in the accumulation of starch in Arabidopsis leaves. Our data also show that ectopic expression of *ESV1* under the control of the *35S* promoter rescues starch granules in the hypocotyl endodermis and root columella of *ESV1* mutant seedlings, but it does not induce the formation of starch granules in other cells (Figure 3C). Even in the endodermis, ectopic *ESV1* rescues starch granules only in the upper hypocotyl endodermis where starch granules are usually present in wild type. Similarly, endodermis-specific expression of *ESV1* under the *SCR* promoter also only rescues starch granules in the upper hypocotyl endodermis but not the endodermis of other regions (Figure 3C).

It is not clear why starch granules form only in the upper hypocotyl endodermis and root columella of dark-grown Arabidopsis seedlings, but there are a few possibilities. First, since starch granules are formed from nutrients stored in seeds, dark-grown seedlings may have insufficient carbon to produce starch granules in other tissues. We noticed, however, that while wild type seedlings still form starch granules only in the upper endodermis and root columella in media supplemented with sucrose, *ESV1* mutant seedlings still lack starch granules (Supplementary Figure 9). This suggests the tissue-specific formation of starch granules cannot be attributed to insufficient carbon. Second, the formation of starch granules in the hypocotyl endodermis and root columella requires plastidic PGM and plastidic AGPase but not plastidic PGI, indicating that glucose 6-phosphate must be transported into plastids to form starch granules in these tissues (Niewiadomski et al., 2005; Kunz et al., 2010; Streb and Zeeman, 2012). Thus, the tissue-specific formation of starch granules could be caused by tissue-specific expression of *glucose 6-phosphate/phosphate translocator* (*GPT*). Previous studies found that two Arabidopsis *GPT* genes (*GPT1, GPT2*) are expressed in all organs including cotyledons, hypocotyls, and roots (Niewiadomski et al., 2005), and *GPT1* is expressed in various parts of roots including the epidermis, vascular tissue, the columella and surrounding cells (Birnbaum et al., 2003; Brady et al., 2007; Tsai et al., 2009). However, since starch granules form only in root columella and not the surrounding cells, it is unlikely that the tissue-specific supply of glucose 6-phosphate is the sole cause of tissue-specific starch granule formation. Third, since starch biosynthesis requires the coordinated action of many enzymes including PGM, AGPase, and SS, tissue-specific starch granule formation may require the entire set of starch biosynthetic genes rather than the tissue-specific expression of any single biosynthetic gene. Indeed, a previous study found that the entire set of starch biosynthetic genes including *PGM, AGPase*, and *SS* are expressed specifically in the root tip but not in other parts of root (Tsai et al., 2009). Thus, although the precise pattern of expression of the starch biosynthetic genes in hypocotyls is unknown, it is tempting to speculate that starch granules form only in the upper hypocotyl endodermis because only the upper hypocotyl endodermis expresses the entire set of starch biosynthetic genes, including the starch granule-stabilizing *ESV1*.

### The Severity of *ESV1* Mutant Phenotypes Is Tissue-Dependent

The starch accumulation patterns in Arabidopsis and rice *ESV1* mutants show similarities and differences. Arabidopsis *ESV1* mutants lack starch granules in the seedling sink tissues: the hypocotyl endodermis and the root columella (Figure 2B). Rice *ESV1* mutants lack starch granules in one sink tissue, the root columella (Figure 7A), but retain loosely packed starch granules in another sink tissue, the grain endosperm (Figure 6D). Both Arabidopsis and rice *ESV1* mutants accumulate starch, in a source tissue, the leaf mesophyll, during the day (Figure 6C, Supplementary Figure 5). These differences in starch accumulation patterns suggest *ESV1* promotes the formation of starch granules differently depending on the plant tissue and species (Supplementary Figure 5, Supplementary Table 1).

It is unclear why starch granules in some tissues are more severely affected by *ESV1* mutation than those in other tissues. It is possible that *ESV1* regulates a tissue-specific metabolic process. Starch is synthesized from glucose 6-phosphate, which is transported into plastids from the cytosol through GPT, via the serial actions of PGM, AGPase, SS, and other synthetic enzymes in Arabidopsis sink tissues (Kofler et al., 2000; Fischer and Weber, 2002; Kunz et al., 2010). In photosynthetic source tissues, however, starch is synthesized from fructose 6-phosphate in the Calvin-Benson cycle via the serial action of plastidic PGI, PGM, AGPase, SS, and other synthetic enzymes (Streb and Zeeman, 2012). Similarly, starch is synthesized from fructose 6-phosphate in rice leaf mesophyll. In cereal endosperms, however, starch is synthesized from ADP-glucose molecules produced in the cytosol and then transported into plastids through BRITTLE 1 (BT1) (Cao et al., 1995; Shannon et al., 1998; Beckles et al., 2001; Jeon et al., 2010; Tuncel and Okita, 2013). Thus, the tissues lacking starch granules in the *ESV1* mutants coincide with tissues using transported glucose 6-phosphate for starch biosynthesis, raising the possibility that *ESV1*'s role in starch granule formation is associated with the transport of glucose 6-phosphate. However, this parallel does not necessarily mean that the role of *ESV1* is associated with the transport of glucose 6-phosphate into plastids. We previously showed starch synthesis in rice pollen relies mainly on glucose 6-phosphate imported into amyloplasts and the reduction of starch synthesis in rice pollen causes male sterility due to reduced pollen viability (Lee et al., 2016). Rice *ESV1* mutants, however, grow normally and bear seeds, indicating that their pollen grains store sufficient starch.

Alternatively, *ESV1* may regulate common metabolic processes found in all tissues, but the severity of the *ESV1* mutant phenotype in a given tissue depends on the ratio of starch synthesis to starch degradation in that tissue. The formation of starch granules is likely to be determined by the balance between starch synthesis and degradation rather than the synthesis alone, as supported by the expression of both synthetic and degrading genes in starch-accumulating tissues (Tsai et al., 2009; Van Harsselaar et al., 2017) or by a high co-expression coefficient for synthetic and degrading genes in deposited transcriptome data (Obayashi et al., 2017). To test this hypothesis, we estimated starch synthetic flow by analyzing the ratio of the expression of *APS1* to *GWD*/*SEX1* mRNAs using reported transcriptome data. Since *APS1* encodes a subunit of an AGPase that is rate-limiting for starch synthesis (Lin et al., 1988; Crevillen et al., 2005) and *GWD* encodes an α-glucan water dikinase required for the first crucial step of starch degradation (Yu et al., 2001; Ritte et al., 2006), the ratio of *APS1* to *GWD* acts as a rough estimate of the balance of starch synthetic flow in different tissues. We found in an analysis of previously reported transcriptome data that Arabidopsis leaves show higher *APS1*/*GWD* ratios than roots (Supplementary Figure 10), suggesting starch synthetic flow is stronger in leaves than roots. Since the Arabidopsis *ESV1* mutation reduces starch accumulation more strongly in roots than in leaves, phenotypic severity is inversely correlated with the strength of starch synthetic flow as represented by the *APS1*/*GWD* ratio. In rice, grains have a higher *APS1*/*GWD* ratio than leaves, which suggests that grains have stronger starch synthetic flow than leaves. Since starch accumulation is more severely reduced in rice leaves than in rice grains, the severity of rice *ESV1* mutant phenotype is also inversely correlated with the *APS1*/*GWD* ratio. Furthermore, Arabidopsis leaves have a higher *APS1/GWD* ratio than rice leaves, suggesting Arabidopsis leaves have stronger starch synthetic flow than rice leaves. Consistent with this, Arabidopsis partitions photo assimilates mainly into transient starch during the day, while rice partitions them mainly into soluble sugar (Lee et al., 2008, 2014). Together, our analysis suggests the severity of the *ESV1* mutation on starch accumulation is inversely associated with tissue-specific biases toward starch synthesis over degradation.

## Methods

### Plant Materials and Growth

Arabidopsis plants (*Arabidopsis thaliana*) were grown in a growth room with a 16-h light/8-h dark cycle at 22–24°C for general growth and seed harvesting. T-DNA insertion mutants for Arabidopsis *ESV1* and *LESV* were obtained from the Arabidopsis stock center and homozygous mutants were selected by genotyping. The corresponding stock numbers are as follows: *ESV1-2* (*GABI_031C11*), *lesv-2* (*Salk_006705*), *lesv-3* (*Salk_006697*), *aps1* (*Salk_040155*), *isa1* (*Salk_117080*), *isa2* (*Salk_029442*). The *phot1 phot2* mutant was obtained from Eiji Gotoh at Kyushu University and the *pgm-1* mutant from Samuel Zeeman at ETH Zurich.

To generate transgenic plants expressing *ESV1*, the *ESV1* coding region was cloned into the *pCAMBIA1300* or its derived vectors containing either GFP or tissue-specific promoters (*ML1* promoter or *SCR* promoter) and introduced into *Arabidopsis* (*35Sp:ESV1*-OX/*ESV1-2, 35Sp:ESV1-GFP, ML1p:ESV1*/*ESV1-2, SCRp:ESV1*/*ESV1-2*). At least two independent transgenic lines were generated. Synthesized *ESV1* genes from *Marchantia polymorpha* (*Mapoly0189s0007*) and *Oryza sativa* (*LOC_Os03g04100*) were also used to generate transgenic lines (*SCRp:*Mp*ESV1*/*ESV1-2, SCRp:*Os*ESV1*/*ESV1-2*). To visualize endodermal plastids, a DNA fragment corresponding to the Rubisco small subunit (RBCS1A) transit peptide was cloned into a binary vector containing the *mCitrine* gene under the control of the *SCR* promoter as previously reported (Saito et al., 2005). All Arabidopsis plants used in this study were of the Columbia ecotype background.

Rice plants (*Oryza sativa*) were grown in a greenhouse with a 14-h light/10-h dark cycle (30/20°C). T-DNA insertion mutants of rice *ESV1* were identified from the rice stock center (Jeon et al., 2000; Jeong et al., 2006). Homozygous mutants were selected by PCR genotyping with gene-specific and T-DNA-specific primers. The corresponding stock numbers are as follows: os*ESV1-1* (*PFG_K-03747*) and os*ESV1-2* (*PFG_1A-07022*). Their parental lines are Kitaake and Dongjin, respectively. The primers used for cloning are described in Supplementary Table 3.

### Identification of Starchless *rgv* Loci

A total of 10,000 seeds from an Arabidopsis transgenic line expressing *PIF1* under the control of the endodermis-specific *SCR* promoter (*SCRp:PIF1*/*pifQ*) were mutagenized with EMS. M_2_ seedlings that did not grow upward in the dark were selected and mutants displaying stable phenotypes over generations were selected as *reduced gravitropic* (*rgv*) mutants. For complementation grouping, seven starchless *rgv* mutants were each crossed with one another and the endodermal starch granules of the F_1_ seedlings were examined with Lugol's iodine. To determine if the starchless mutants are allelic to known starchless biosynthetic mutants, *rgv1-1, rgv2-1*, and *rgv3-1*, representing three complementation groups, were crossed with *pgm, aps1, isa1*, and *isa2* mutants and the endodermal starch granules of the resulting F_1_ seedlings were examined with Lugol's iodine. Simultaneously, the genomic regions of *PGM, APS1*, and *ISA1* were amplified and sequenced from the seven starchless mutants. To identify the mutated loci in the three *rgv1* mutant alleles (*rgv1-1, rgv1-2, rgv1-3*), each mutant was backcrossed to the parental line three times and then used for whole genome sequencing analysis. The whole-genome sequencing was performed by Illumina NextSeq and the results were mapped to the Arabidopsis reference genome (Arabidopsis.org; TAIR10). High quality homozygous single nucleotide polymorphisms (SNPs) caused by G to A or C to T substitutions were identified and, by comparing the SNPs in the three *rgv1* alleles with the parental line, SNPs were identified in *AT1G42430* in all three *rgv1* alleles but not in the parental line. To further support the hypothesis that *rgv1* mutants are caused by mutations in this gene, a T-DNA insertion line of *AT1G42430* was obtained from the Arabidopsis stock center. After confirming that the *rgv1* mutants were caused by mutations in *AT1G42430*, the *rgv1* mutant was renamed to the *ESV1* mutant.

### Lugol's Iodine Staining of Starch Granules

Lugol's iodine staining was used to visualize starch granules in seedlings. Arabidopsis seedlings were fixed in FAA solution (5% Formaldehyde, 5% Acetic acid, 45% EtOH) for 24 h at 4°C. After fixation, the seedlings were washed with 50% ethanol once, and stained with I_2_-KI solution (2% w/v Iodine, 5% w/v Potassium iodide, 20% v/v Trichloroacetic acid) for 1 min. Seedlings were de-stained with a clearing solution (Trichloroacetic acid: phenol: lactate = 2: 1: 1) for 1 min and mounted on slide glass with fresh clearing solution for microscopy. For rice, samples were fixed in 0.05 M sodium-phosphate buffer (pH 7.4) containing 2% paraformaldehyde for 4 h at 4°C. After fixation, the tissues were washed overnight in phosphate-buffered saline (PBS, pH 7.4) containing 6.8% sucrose, dehydrated in an ethanol gradient series, and embedded in Technovit 8100 resin (Kulzer, Frankfurt, Germany). Tissues were then sliced into 10 μm sections with a rotary microtome (RM2165; Leica, Wetzlar, Germany) and stained with 10% diluted I_2_-KI solution (Sigma-Aldrich, St. Louis, MO, USA) for 1 min.

### Seedling Gravitropism

Hypocotyl negative gravitropism was determined by growing seedlings for 3 days on horizontal plates in different light conditions and counting the number of standing seedlings. For the assay, surface-sterilized seeds were plated on half Murashige and Skoog (MS) media (1/2 MS, 0.8% phytoagar, and 0.05% MES, pH 5.7) and stratified for 3 days in the dark at 4°C. After the stratification, the seeds were incubated under white light (50 μmol/m^2^s) for 6 h at 22°C to induce germination. Germination-induced seeds were then incubated for 3 days in the dark or in red light (20 μmol/m^2^s) at 22°C. Seedlings were counted as standing if their cotyledons did not touch the agar surface. Total 40 of seedlings were used for each experiment (*n* = 3, biological replicates). Phototropic seedlings were similarly determined after growing them on horizontal plates under blue light (1 μmol/m^2^s) for 4 days. To determine Arabidopsis root gravitropic responses, seedlings were grown for 5 days on vertical plates under white light (50 μmol/m^2^s). Then, they were grown for an additional 12 h after rotating the plates by 90 degrees. The angles formed by the bent root were measured and supplementary angles were used to indicate the degree of root gravitropism. To determine rice root gravitropic responses, surface-sterilized seeds were germinated on half MS media (1/2 MS, 3% phytoagar, pH 5.7) in Magenta vessels (Sigma-Aldrich, St. Louis, MO, USA) for 7 days in the dark at 25°C until the shoots and roots established a vertical extension of seedling growth. These were then further incubated for 1 day after rotating the vessels by 90 degrees.

### Sedimentation of Endodermal Plastids

Seedlings were grown on vertical plates in the dark for 3 days before being inverted. For the *ESV1* mutant, seedlings were rearranged vertically at day 2 under a safety green light and allowed to grow 1 more day in the dark before inversion. At the indicated times after inversion, the seedlings were decapitated with a blade, transferred to 4% paraformaldehyde + 0.1% triton X-100 solution, fixed under vacuum for 1 h, and washed twice with PBS buffer. The seedlings were then transferred to 0.1% Calcofluor white solution for 1 h to stain the cell walls (Fluorescent Brightener 28, MP Biomedicals). After two more washes with PBS buffer, the seedlings were mounted on a slide glass with anti-fade mounting solution (VECTASHIELD Antifade Mounting Medium H-1000). Fluorescence images were taken with a confocal microscope (LSM780, ZEISS). The relative position of each plastid was calculated by assigning the initial bottom of a cell to 0 and the top of the cell to 100.

### Protein and mRNA Expression Analysis

After the induction of seed germination, seedlings were grown on half MS media with 1% sucrose either in the dark or in red light (20 μmol/m^2^s) for 3 days and whole seedlings were harvested for RNA or protein expression analysis. For mRNA expression analysis, total RNAs were extracted using the Spectrum plant total RNA kit (Sigma-Aldrich) and converted to cDNA using MMLV-RTase (Promega). Relative transcript levels were determined by quantitative PCR (CFX connect, Bio-rad) and normalized to the level of *PP2A*. All experiments were performed with biological triplicates.

For protein expression analysis, harvested seedlings were ground in liquid nitrogen and further homogenized in a denaturing buffer (100 mM NaH_2_PO_4_, pH 8.0, 10 mM Tris-Cl, 8 M Urea, 1 mM phenylmethylsulfonyl fluoride (PMSF), and protease inhibitor cocktail). After removing the debris by centrifugation, the supernatant protein extract was mixed with 5X SDS sample buffer [50 mM Tris-Cl, pH 6.8, 10% glycerol, 2% sodium dodecyl sulfate (SDS), 0.05% bromophenol blue, and 100 mM dithiothreitol (DTT)] and boiled at 100°C for 5 min. Proteins were then detected by immunoblot analysis using an anti-GFP antibody for *ESV1*-GFP and an anti-α-tubulin antibody for α-tubulin. For the quantitative analysis, the intensity of *ESV1*-GFP was normalized by α-tubulin (*n* = 3, biological replicates).

### Determination of Soluble Sugars and Starch

Approximately 100 mg of leaves were sampled from 6-week-old rice plants grown under a 14/10 light cycle in a greenhouse. Each sample was harvested at zeitgeber (ZT) 0, 8, 16, 18, 20, and 22. Glucose, fructose, sucrose, and starch were measured in the soluble and insoluble fractions of ethanol-water extracts using enzymatic methods (Lee et al., 2005). The measured metabolite contents were normalized to fresh weights (*n* = 3, biological replicates).

### Scanning Electron Microscopy

Scanning Electron Microscope (SEM) analysis were carried out with Carl Zeiss MERLIN™ FE-SEM operated at low accelerating voltage of 1 kV. The fractured surface morphologies of wild type and mutant rice grains were observed without any coating materials.

## Accession Numbers

### Arabidopsis Thaliana

*APS1*: *AT5G48300, GWD*: *AT1G10760, IAA19: AT3G15540, ISA1*: *AT2G39930, ISA2*: *AT1G03310, ML1*: *AT4G21750, PGM*: *AT5G51820, PHOT1*: *AT3G45780, PHOT2*: *AT5G58140, PHYA*: *AT1G09570, PHYB*: *AT2G18790, PIF1*: *AT2G20180, PIF3*: *AT1G09530, PIF4*: *AT2G43010, PIF5*: *AT3G59060, PIL1*: *AT2G46970, PP2A*: *AT1G13320, RBCS1A*: AT1G67090, *ESV1*: *AT1G42430, LESV*: *AT3G55760, and SCR*: *AT3G54220*.

### Oryza Sativa

Os-*ESV1*: *LOC_Os03g04100, APS1*: *LOC_Os09g12660, GWD*: *LOC_Os06g30310, APS2: LOC_Os08g25734, APL2: LOC_Os01g44220, PGM: LOC_Os10g11140, APL4: LOC_Os07g13980*.

### Marchantia Polymorpha

Mp-*ESV1*: *Mapoly0189s0007*.

## Data Availability Statement

The raw data supporting the conclusions of this article will be made available by the authors, without undue reservation.

## Author Contributions

KS, D-WL, JeK, J-SJ, and GC designed the experiments. KS, D-WL, JeK, JaK, HG, and KK performed the experiments. KS, D-WL, J-SJ, and GC wrote the paper. All authors contributed to the article and approved the submitted version.

## Conflict of Interest

The authors declare that the research was conducted in the absence of any commercial or financial relationships that could be construed as a potential conflict of interest.

## Publisher's Note

All claims expressed in this article are solely those of the authors and do not necessarily represent those of their affiliated organizations, or those of the publisher, the editors and the reviewers. Any product that may be evaluated in this article, or claim that may be made by its manufacturer, is not guaranteed or endorsed by the publisher.
