# Supplementary material for: EARLY STARVATION 1 Is a Functionally Conserved Protein Promoting Gravitropic Responses in Plants by Forming Starch Granules
Source: Front Plant Sci. 2021 Jul 23;12:628948. doi: 10.3389/fpls.2021.628948 (PMC8343138; doi:10.3389/fpls.2021.628948)
Supplement: Supplementary file 1 [file Data_Sheet_1.PDF]

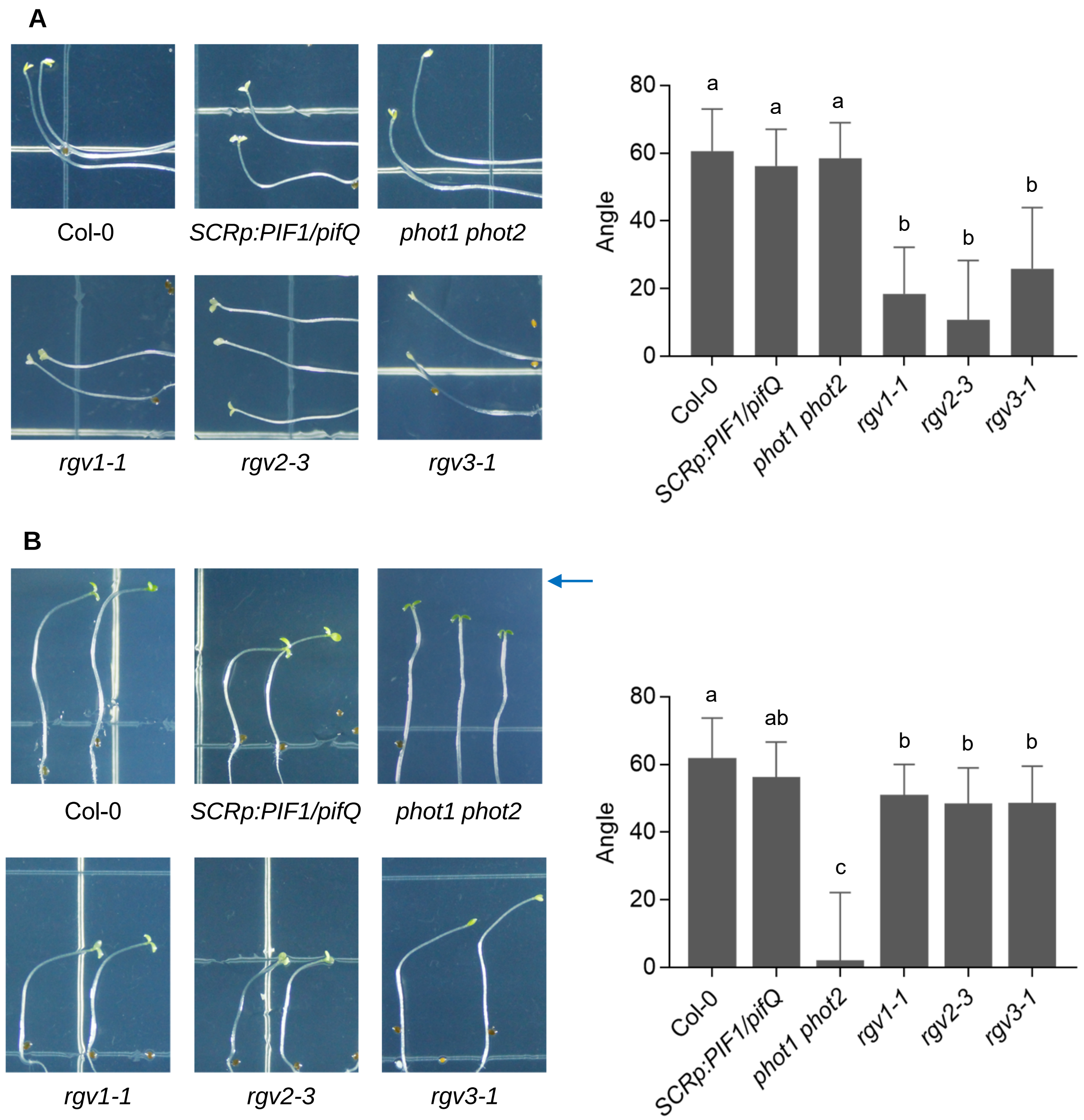

Supplemental Figure 1. Hypocotyl bending of *rgv* mutants toward the direction of gravity or blue light.

(A) Shoot gravitropic responses of *rgv* mutants. Arabidopsis seedlings vertically grown on MS-agar plates for three days in the dark were rotated by 135 degrees and grown two more days before taking images and measuring bending angles. The graph in the right panel indicates quantitation of bending angles. Letters indicate statistical significance determined by an ANOVA with Tukey's HSD post-hoc test for multiple comparisons ($p<0.01$). Error bars=SD ($n>40$).

(B) Shoot phototropic responses of *rgv* mutants. Arabidopsis seedlings vertically grown on MS-agar plates for three days in the dark were irradiated with blue light (1 μmol/m^2s) from a side (blue arrow) and grown two more days before taking images and measuring bending angles. The graph in the right panel indicates quantitation of bending angles. Letters indicate statistical significance determined by an ANOVA with Tukey's HSD post-hoc test for multiple comparisons ($p<0.01$). Error bars=SD ($n>40$).
