# Supplementary material for: EARLY STARVATION 1 Is a Functionally Conserved Protein Promoting Gravitropic Responses in Plants by Forming Starch Granules
Source: Front Plant Sci. 2021 Jul 23;12:628948. doi: 10.3389/fpls.2021.628948 (PMC8343138; doi:10.3389/fpls.2021.628948)
Supplement: Supplementary file 2 [file Data_Sheet_2.PDF]

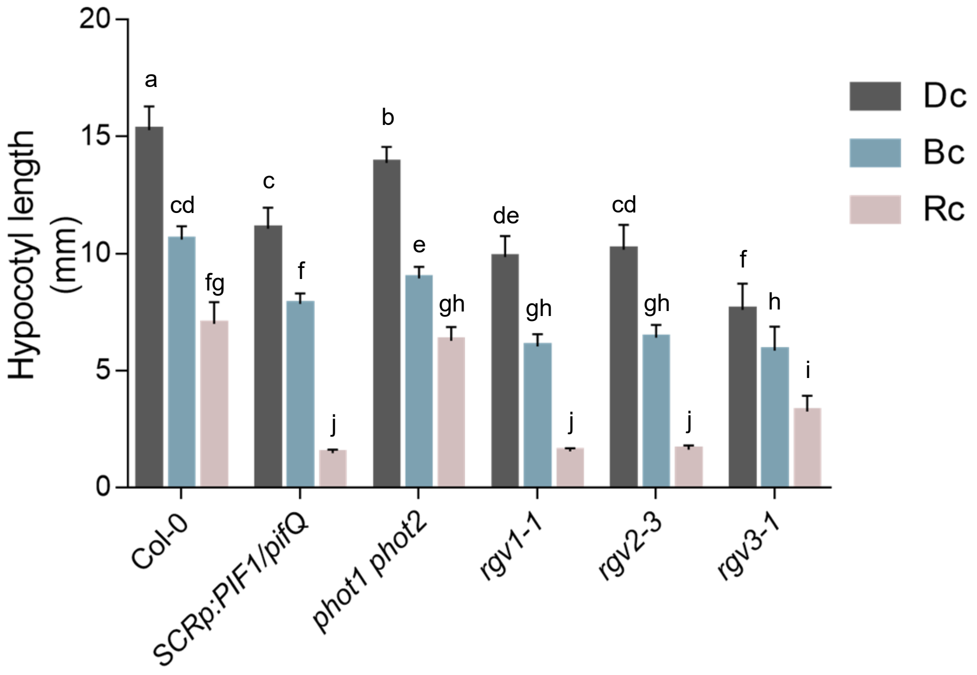

Supplemental Figure 2. Hypocotyl lengths of *rgv* mutants.
Hypocotyl lengths of 4-d-old seedlings grown in the dark (Dc), blue light (Bc; 1 μmol/m^{2}s), or red light (Rc; 20 μmol/m^{2}s) were measured. Letters indicate statistical significance determined by an ANOVA with Tukey's HSD post-hoc test for multiple comparisons ($p<0.01$). Error bars=SEM ($n = 15$).
