# Supplementary material for: EARLY STARVATION 1 Is a Functionally Conserved Protein Promoting Gravitropic Responses in Plants by Forming Starch Granules
Source: Front Plant Sci. 2021 Jul 23;12:628948. doi: 10.3389/fpls.2021.628948 (PMC8343138; doi:10.3389/fpls.2021.628948)
Supplement: Supplementary file 3 [file Data_Sheet_3.PDF]

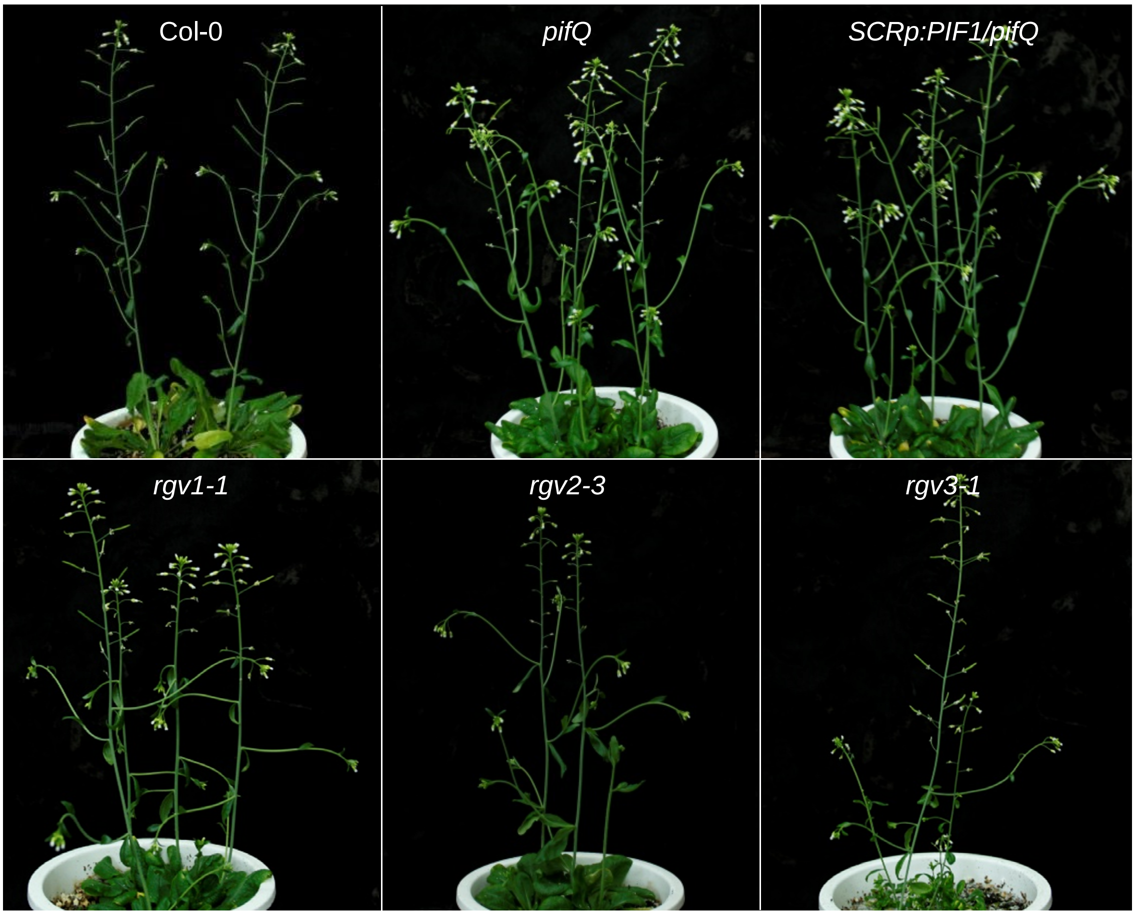


**Supplemental Figure 3. Three starchless *rgv* mutants develop branches with wide angles.**

All plants were grown in a growth room at 22°C in a 16h/8h light/dark cycle and these images were taken 4–5 weeks after seeding. Col-0: wild type, *pifQ*: *pif1 pif3 pif4 pif5* mutant, *SCRp:PIF1/pifQ*: parental line expressing *PIF1* under the *SCR* promoter in the *pifQ* mutant background. *rgv1-1*, *rgv2-3*, and *rgv3-1* represent the three starchless complementation groups.
