# Supplementary material for: EARLY STARVATION 1 Is a Functionally Conserved Protein Promoting Gravitropic Responses in Plants by Forming Starch Granules
Source: Front Plant Sci. 2021 Jul 23;12:628948. doi: 10.3389/fpls.2021.628948 (PMC8343138; doi:10.3389/fpls.2021.628948)
Supplement: Supplementary file 4 [file Data_Sheet_4.PDF]

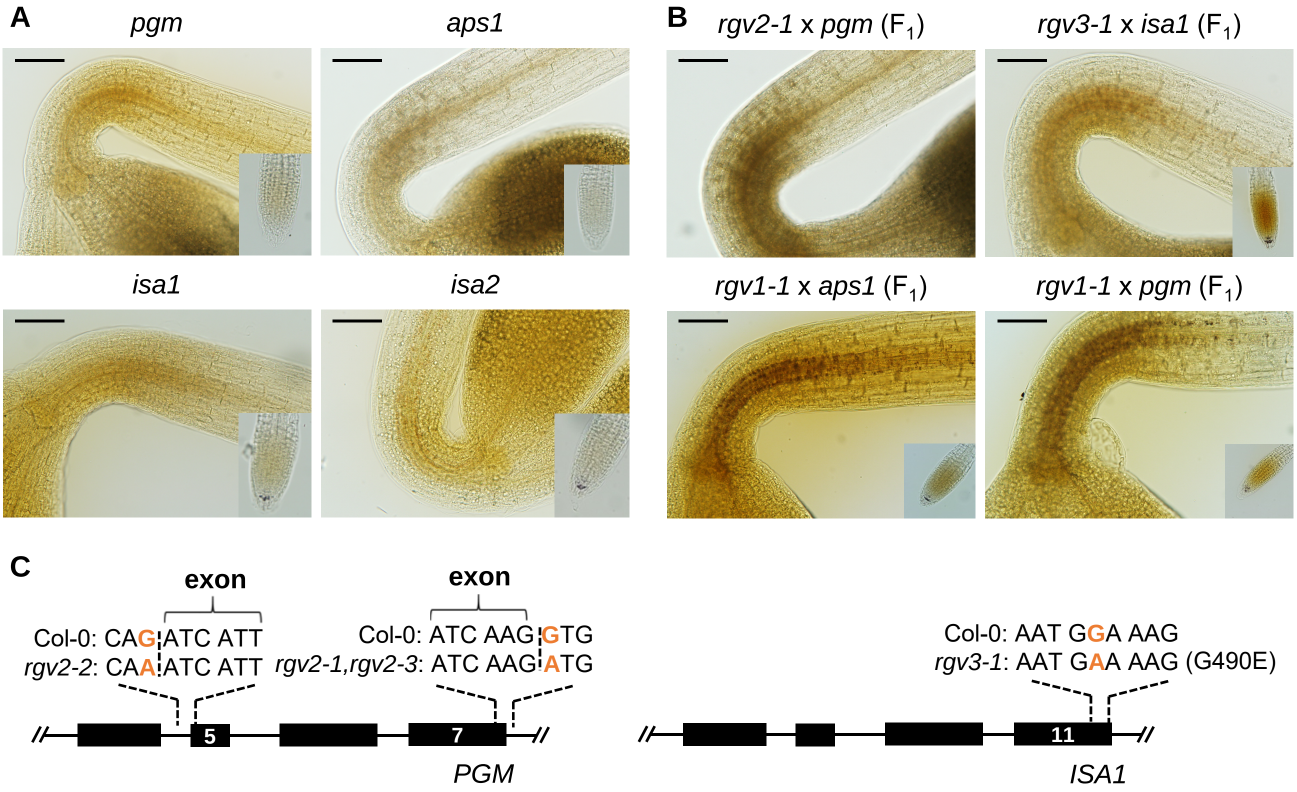

Supplemental Figure 4. *rgv2* and *rgv3* mutants have mutations in *PGM* and *ISA1*, respectively.

(A) Starchless phenotypes in four starch biosynthetic mutants. Starch granules were visualized by staining 3-day-old dark-grown mutant seedlings with Lugol's iodine. Insets show starch granules in root columella. *pgm*: *pgm-1*, *aps1*: *Salk_040155*, *isa1*: *Salk_117080*, *isa2*: *Salk_029442*. Scale bar = 100μm.

(B) Complementation test between the three starchless *rgv* mutants and these starch biosynthetic mutants. The three *rgv* mutants were crossed with *pgm*, *aps1*, and *isa1* and the starch granules in 3-day-old dark-grown F1 progeny were visualized by staining with Lugol's iodine. Scale bar = 100μm.

(C) Mutation loci for the *rgv2* and *rgv3* mutants. The genomic regions of the starch biosynthetic genes from three *rgv* mutants were amplified and sequenced. Partial gene structures of *PGM* and *ISA1* as well as the nucleotide sequences near each mutated locus (red-colored) are shown for the *rgv2* and *rgv3* mutant alleles. Black bars: exons, Lines: introns, Numbers in black bars: exon number.
