# Supplementary material for: EARLY STARVATION 1 Is a Functionally Conserved Protein Promoting Gravitropic Responses in Plants by Forming Starch Granules
Source: Front Plant Sci. 2021 Jul 23;12:628948. doi: 10.3389/fpls.2021.628948 (PMC8343138; doi:10.3389/fpls.2021.628948)
Supplement: Supplementary file 5 [file Data_Sheet_5.PDF]

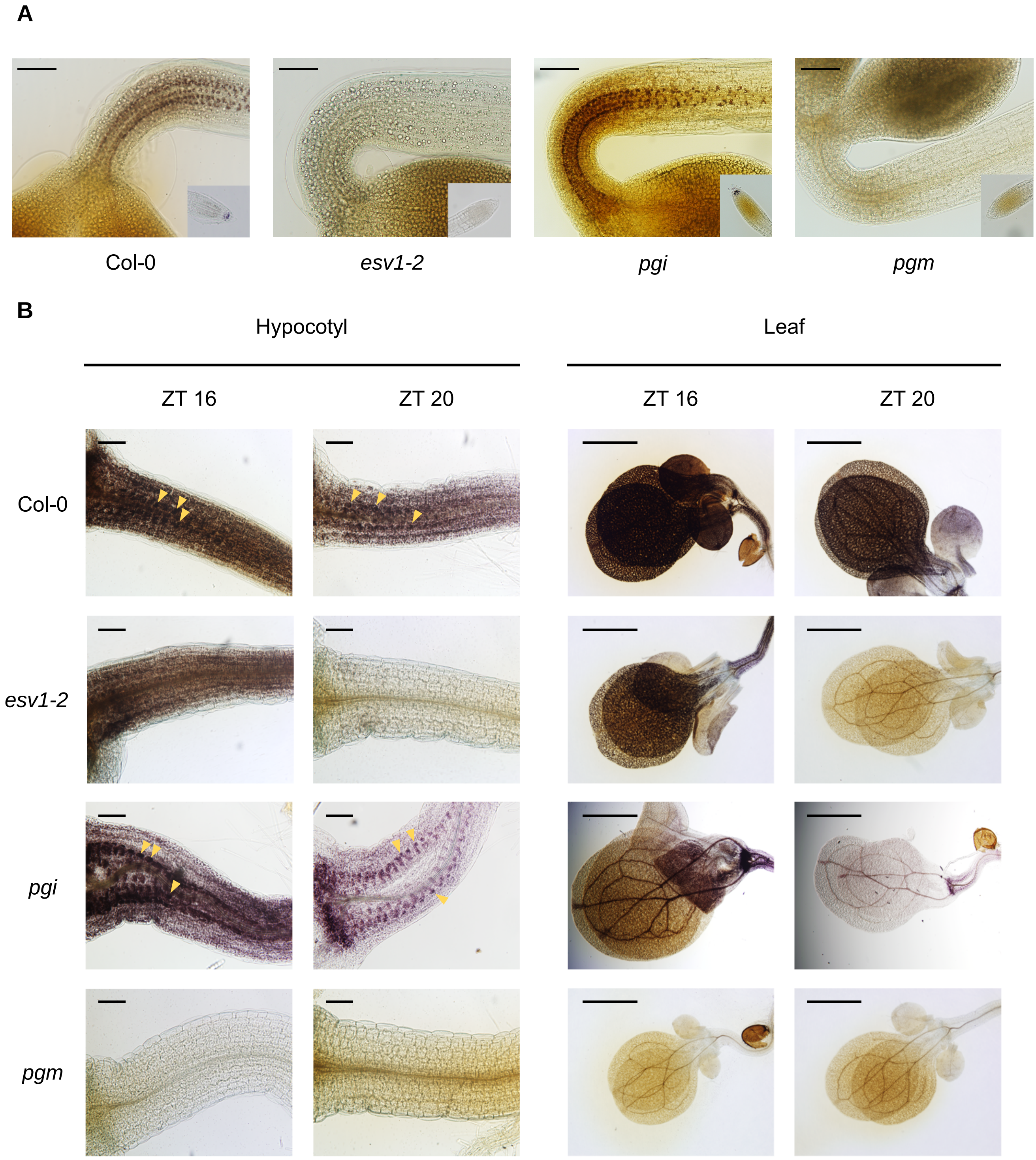

Supplemental Figure 5. The *esv1* mutation reduces the starch granule formation preferentially in hypocotyl endodermis.
(A) The lack of hypocotyl endodermal starch granules in the etiolated *esv1* mutant but not in the *pgi* mutant. Endodermal starch granules were visualized by staining 3-day-old dark-grown seedlings with Lugol's iodine. Col-0: wild type, *esv1-2*: a T-DNA insertion mutant in *AT1G42430* (*GABI-031C11*), *pgi*: *pgi1-1*, *pgm*: *pgm-1*. Scale bar = 100µm.
(B) The lack of hypocotyl endodermal starch granules in the light-grown *esv1* mutant but not in the *pgi* mutant. The 7-day-old light-grown (16h-light/8h-dark) seedlings were sampled at ZT16, ZT20. After removing chlorophyll by ethanol, starch granules in hypocotyl (left), and leaf (right) were visualized by staining with Lugol's iodine. Yellow arrow heads indicate starch granules in hypocotyl endodermis. Scale bar = 100µm (left), 1mm (right).
