# Supplementary material for: EARLY STARVATION 1 Is a Functionally Conserved Protein Promoting Gravitropic Responses in Plants by Forming Starch Granules
Source: Front Plant Sci. 2021 Jul 23;12:628948. doi: 10.3389/fpls.2021.628948 (PMC8343138; doi:10.3389/fpls.2021.628948)
Supplement: Supplementary file 6 [file Data_Sheet_6.PDF]

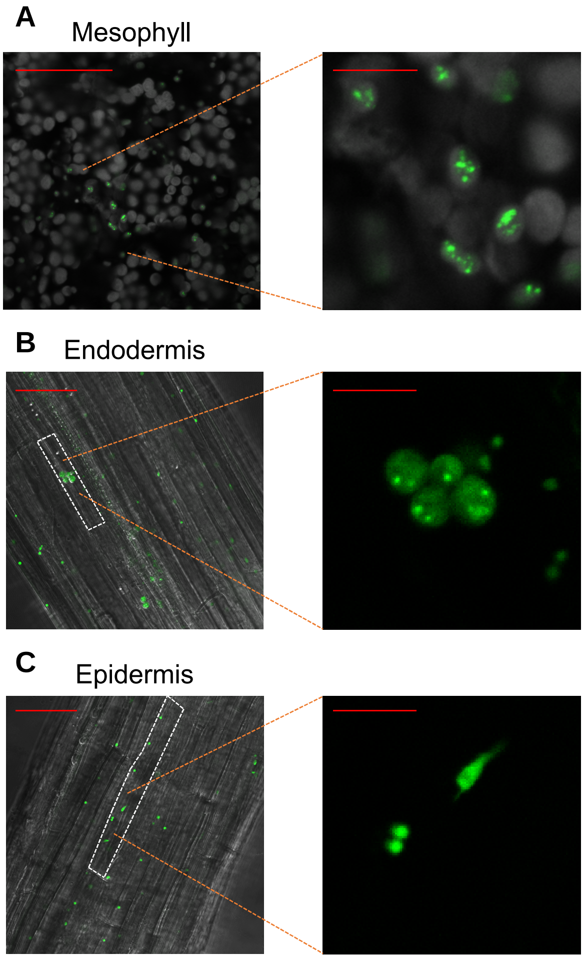

Supplemental Figure 6. Punctate ESV1-GFP signal in starch-accumulating plastids. ESV1-GFP-positive puncta in light-grown cotyledon mesophyll chloroplasts **(A)**, dark-grown hypocotyl endodermal amyloplasts **(B),** and diffuse ESV1-GFP signal in dark-grown hypocotyl epidermal plastids **(C)**. The grey discs in **(A)** are chloroplasts emitting chlorophyll fluorescence. The white dotted line in the left panels indicates a single cell and the right panels are magnified views of its plastids. ESV1-GFP signals were observed by confocal microscopy. Scale bar = 50μm(left), 10μm(right), respectively .
