# Supplementary material for: EARLY STARVATION 1 Is a Functionally Conserved Protein Promoting Gravitropic Responses in Plants by Forming Starch Granules
Source: Front Plant Sci. 2021 Jul 23;12:628948. doi: 10.3389/fpls.2021.628948 (PMC8343138; doi:10.3389/fpls.2021.628948)
Supplement: Supplementary file 7 [file Data_Sheet_7.PDF]

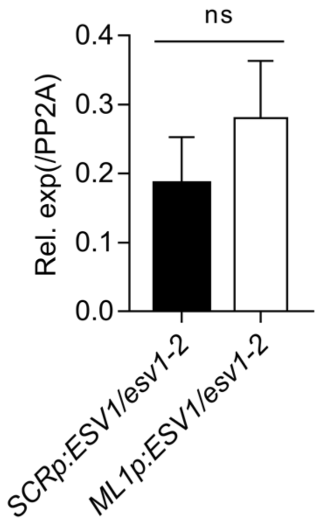

Supplemental Figure 7. Endodermis- and epidermis-specific *ESV1* lines express similar levels of transgenic *ESV1* mRNA.

Three-day-old dark-grown transgenic seedlings (*SCRp:ESV1*/*esv1-2*, *ML1p:ESV1*/*esv1-2*) were used for mRNA expression analysis. *SCRp:ESV1*/*esv1-2*: transgenic plants expressing *ESV1* under the endodermis-specific *SCR* promoter in the *esv1-2* mutant background, *ML1p:ESV1*/*esv1-2*: transgenic plants expressing *ESV1* under the epidermis-specific *ML1* promoter in the *esv1-2* mutant background. ns: non-significant ($p<0.05$, Student's t-test), Error bars=SEM (n=3 biological replicates).
