# Supplementary material for: EARLY STARVATION 1 Is a Functionally Conserved Protein Promoting Gravitropic Responses in Plants by Forming Starch Granules
Source: Front Plant Sci. 2021 Jul 23;12:628948. doi: 10.3389/fpls.2021.628948 (PMC8343138; doi:10.3389/fpls.2021.628948)
Supplement: Supplementary file 8 [file Data_Sheet_8.PDF]

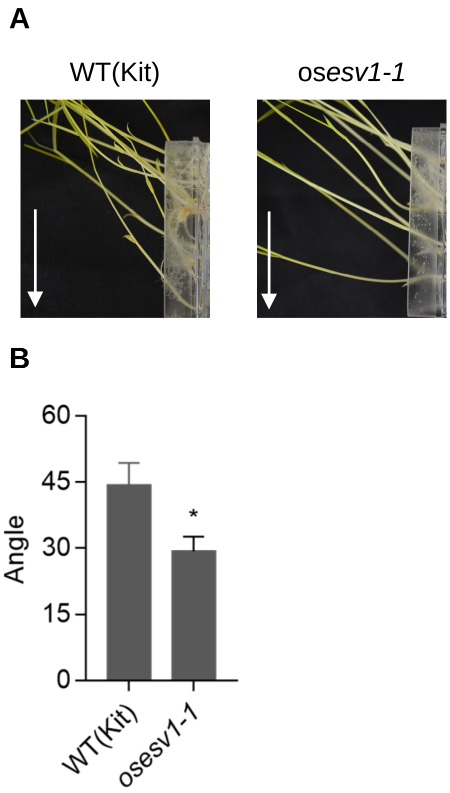

Supplemental Figure 8. Rice *esv1* mutant displays reduced shoot gravitropism.
(A) Reduced shoot gravitropic response of rice *esv1* mutant. Wild type (Kit) and rice *esv1* mutant (*osesv1-1*) seedlings grown on MS-agar for five days in the dark were rotated by 90 degrees and grown one more day before taking images and measuring bending angles. Arrows indicate the direction of gravity after the rotation.
(B) Quantitation of bending angles of wild type and rice *esv1* mutant as in **(A)**. An asterisk indicates a significant difference from wild type (*, $p<0.05$; Student's t-test). Error bars=SD (n=10).
