# Supplementary material for: EARLY STARVATION 1 Is a Functionally Conserved Protein Promoting Gravitropic Responses in Plants by Forming Starch Granules
Source: Front Plant Sci. 2021 Jul 23;12:628948. doi: 10.3389/fpls.2021.628948 (PMC8343138; doi:10.3389/fpls.2021.628948)
Supplement: Supplementary file 9 [file Data_Sheet_9.PDF]

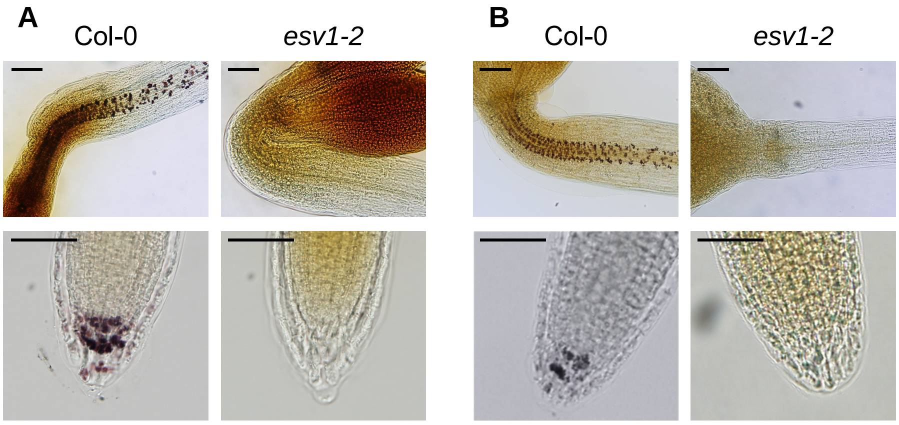

Supplemental Figure 9. Sucrose supplementation does not change the tissue-specific formation of starch granules in wild type seedlings nor does it restore starch granules in the *esv1* mutant.
Starch granules in the upper hypocotyl endodermis (upper) and root columella (lower) were visualized by staining 3-day-old dark-grown seedlings with Lugol’s iodine. Seedlings were grown in MS media either with **(A)** or without **(B)** the addition of 1% sucrose. Scale bar = 50µm.
