# Supplementary material for: EARLY STARVATION 1 Is a Functionally Conserved Protein Promoting Gravitropic Responses in Plants by Forming Starch Granules
Source: Front Plant Sci. 2021 Jul 23;12:628948. doi: 10.3389/fpls.2021.628948 (PMC8343138; doi:10.3389/fpls.2021.628948)
Supplement: Supplementary file 10 [file Data_Sheet_10.PDF]

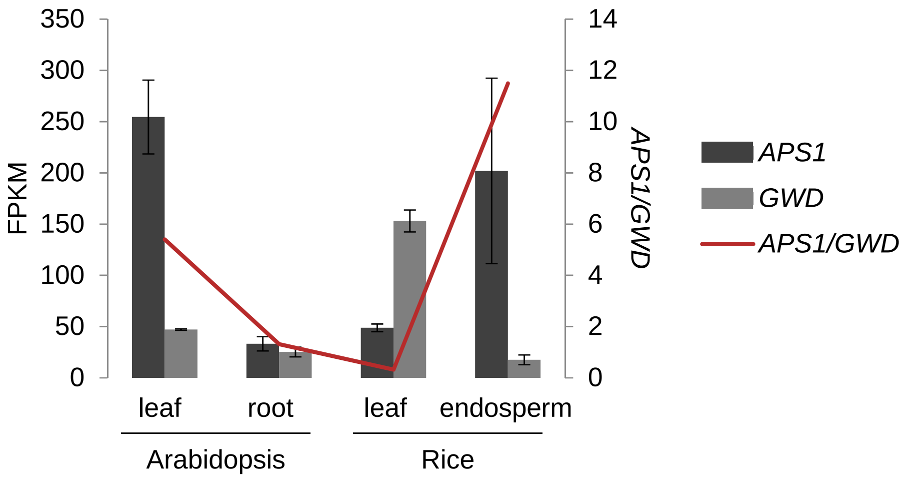

Supplemental Figure 10. The phenotypic severities of *esv1* mutants are inversely correlated with the strength of starch synthetic flow in different tissues.

The ratio of *APS1* to *GWD* mRNA levels are used as an estimate of the strength of starch synthetic flow. *APS1* and *GWD* mRNA levels were extracted from previously reported RNA-seq data deposited in the public GEO Database (Arabidopsis leaf: GSE128434, Arabidopsis root: GSE87880, GSE122355, Rice leaf: GSE128434, Rice endosperm: GSE73220) and are presented as Fragments Per Kilobase of transcript per Million (FPKM). Average FPKM values are plotted on the left axis. Error bars=SEM (n=2). FPKM ratios of *APS1* to *GWD* (*APS1/GWD*) are plotted on the right axis. For the analysis, raw read data from Arabidopsis leaf (SRR8742291, SRR8742303), Arabidopsis root (SRR4418017, SRR8178031), Rice leaf (SRR8742300, SRR8742312), and Rice endosperm (SRR2338866, SRR2338867) were mapped to their respective reference genomes, (Arabidopsis: TAIR10, Rice: IRGSP-1.0) using TopHat2 and the FPKM was calculated using cufflinks. *APS1* (*AT5G48300, LOC_Os09g12660;* Arabidopsis and rice, respectively) and *GWD* (*AT1G10760, LOC_Os06g30310*; Arabidopsis and rice, respectively).
