# Supplementary material for: EARLY STARVATION 1 Is a Functionally Conserved Protein Promoting Gravitropic Responses in Plants by Forming Starch Granules
Source: Front Plant Sci. 2021 Jul 23;12:628948. doi: 10.3389/fpls.2021.628948 (PMC8343138; doi:10.3389/fpls.2021.628948)
Supplement: Supplementary file 11 [file Data_Sheet_11.PDF]

Supplemental Table 1. The comparison of Arabidopsis and rice *esv1* mutant phenotypes.

***esv1* mutant phenotype**	**Arabidopsis**	**Rice**
Leaf	Normal	Reduced
Root tip	Absent	Absent
Hypocotyl endodermis	Absent	-
Grain	-	Reduced<br>Loosely packed granules
