# Supplementary material for: EARLY STARVATION 1 Is a Functionally Conserved Protein Promoting Gravitropic Responses in Plants by Forming Starch Granules
Source: Front Plant Sci. 2021 Jul 23;12:628948. doi: 10.3389/fpls.2021.628948 (PMC8343138; doi:10.3389/fpls.2021.628948)
Supplement: Supplementary file 12 [file Data_Sheet_12.PDF]

Supplemental Table 2. Statistical analysis table for Figure 6C. . Asterisks indicate significant differences from the respective wild type (*, $p<0.05$; Student's t-test). ns indicates no significant difference.

	Starch		Sucrose		Glucose		Fructose	
ZT (h)	WT(kit) vs *osesv1-1*	WT(DJ) vs *osesv1-2*	WT(kit) vs *osesv1-1*	WT(DJ) vs *osesv1-2*	WT(kit) vs *osesv1-1*	WT(DJ) vs *osesv1-2*	WT(kit) vs *osesv1-1*	WT(DJ) vs *osesv1-2*
0	ns	ns	ns	ns	ns	ns	ns	ns
8	*	*	ns	*	ns	ns	ns	ns
16	*	*	ns	*	*	ns	ns	ns
18	*	*	*	ns	ns	*	ns	ns
20	*	*	ns	*	ns	ns	ns	ns
22	*	*	ns	*	ns	ns	ns	ns
24	ns	ns	ns	ns	ns	ns	ns	ns
