# Supplementary material for: EARLY STARVATION 1 Is a Functionally Conserved Protein Promoting Gravitropic Responses in Plants by Forming Starch Granules
Source: Front Plant Sci. 2021 Jul 23;12:628948. doi: 10.3389/fpls.2021.628948 (PMC8343138; doi:10.3389/fpls.2021.628948)
Supplement: Supplementary file 13 [file Data_Sheet_13.PDF]

Supplemental Table 3. List of pirmers used in this study.

Cloing & Genotyping	
Primer	Sequence (5' → 3')
ESV1 5' XbaI	GAGTCTAGAATGAGCGAAATGGCGGCTAG
ESV1 3' BamHI	GAGGGATCCGTTGTGGTTGGTCAGGGGG TA
ESV1 3' BamHI stop	GAGGGATCCGTCATTGTGGTTGGTCAGGG GGTA
RGVH 5' xbaI	GAGTCTAGAATGGCTTTGCGTTTAGGTGT
RGVH 3' BamHI	GAGGGATCCGGGACATATCAGAAGGCTTC T
RGVH 3' BamHI with stop	GAGGGATCCCCTAGGACATATCAGAAGGC T
At_T-DNA specific primer	ATTTTGCCGATTTCGGAAC
osesv1-1 genotyping Lp	CTGACACTATCAAAATTTAATCCCACCAA
osesv1-1 genotyping Rp	GGTTTATTCCACTACATAAAGCGTGTGTC
osesv1-2 genotyping Lp	GTGGCACTAGTTAGAATCGAAGAT
osesv1-2 genotyping Rp	AGAACAAGTTTCCTTCAAACAAAG
Os_T-DNA specific primer	ATCCAGACTGAATGCCCACAGG
RbcSF	GGAG CCCGGG CAAAACAAAAA ATGGCTTCCT CTATGCTCTC
RbcSR	CCTCGGATCCACCTTCGGAATCGGTAAGG TCAG
RT-qPCR	
ESV1 qPCR F	GACATAGTGGTGGATGAAGAAACATAC
ESV1 qPCR R	AAACTCGAGGTTTGGGTAGACAC
PIL1 qPCR F	GCGAAGACACAAGTTCATGCGAGA
PIL1 qPCR R	CTGCTGCACCCATATGCATTCCT
PP2A qPCR F	CATTGTAGAACTTGCTGAAGACAGG
PP2A qPCR R	CAAGACGCTTCAGATTGTTTGCAGC
